# Negative Electric Vehicle Emissions: Vehicle-to-Grid Can Incentivize Enough Wind and Solar Investment to Reverse EV Charging Emissions

**DOI:** 10.1021/acs.est.5c06944

**Published:** 2025-09-27

**Authors:** Jiahui Chen, Michael T. Craig, Jeremy Michalek, Matthew Bruchon, Parth Vaishnav

**Affiliations:** † School for Environment and Sustainability, 1259 University of Michigan, Ann Arbor, Michigan 48109, United States; ‡ Department of Engineering and Public Policy, 6612 Carnegie Mellon University, Pittsburgh, Pennsylvania 15213, United States; § Department of Industrial and Operations Engineering, 1259 University of Michigan, Ann Arbor, Michigan 48109, United States; ∥ Department of Mechanical Engineering, 6612 Carnegie Mellon University, Pittsburgh, Pennsylvania 15213, United States; ⊥ Department of Civil and Environmental Engineering, 6612 Carnegie Mellon University, Pittsburgh, Pennsylvania 15213, United States

**Keywords:** electric vehicles, energy storage, renewable energy, climate change, air emissions, greenhouse gas emissions, externalities

## Abstract

Consequential emission analyses of power system interventions typically overlook induced structural changes. We estimate the consequential power system emission effects of plug-in electric vehicle (PEV) charging due to both operational effects and induced renewable capacity investments. We study PJM, the largest U.S. grid operator, with a grid mix representative of North America, assuming 10% fleet electrification. We find that charging immediately after each day’s final trip increases grid emission externalities and provides little incentive to increase renewable infrastructure investment. In contrast, flexible cost-minimizing charging creates economic incentives to increase wind and solar capacity investment by 4% for unidirectional charging or by 23% for bidirectional vehicle-to-grid (V2G). This induced investment reverses emission consequences of PEV charging: estimates ignoring capacity changes find PEVs increase grid emission externalities by $240–$610 per PEV-year, whereas estimates accounting for induced renewable investments find that adding PEV load can reduce total grid emission externalities by $230 per PEV-year with flexible charging and by $2200 per PEV-year with V2G. Overall, results suggest that leveraging flexibility in charge timing and V2G to reduce power system costs can also produce substantial emission cobenefits.

## Graphical abstract

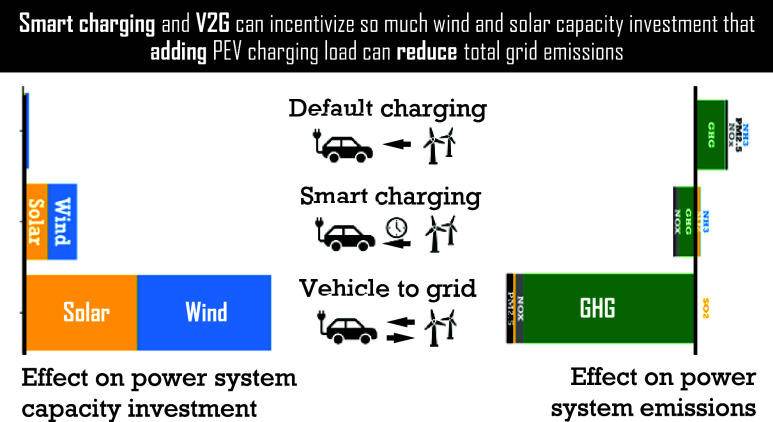

## 1 Introduction

Plug-in electric vehicles (PEVs) and wind and solar power (WSP) are central to global climate efforts. In 2023, global PEV sales exceeded 14 million, or 15.8% of new cars, up from 2.6% in 2019,^1^ with growth expected to continue due to declining costs and supportive policies in many countries.^1,2^ WSP capacity has also expanded rapidly, driven by similar trends;^3,4^ in the U.S., WSP generated 14% of electricity in 2022, up from 2% in 2010.

However, growing PEV penetration and WSP penetration can challenge grid reliability,^5,6^ and high WSP penetrations may depress electricity prices, reducing renewable generator revenues.^7–9^ Load flexibility—especially via smart PEV charging and vehicle-to-grid (V2G) technology—can help address these issues and influence the emissions impact of rising PEV use.^10–14^

Most prior studies estimating the impact of PEV charging on power systems treat infrastructure as fixed, capturing only short-run operational effects of added PEV load, as summarized in the top row of Table 1.^15–25^ Gagnon et al.^26^, Holland et al.^27^ and Hanig et al.^100^ critique this approach, noting it overlooks how load increase could drive new generation capacity, some of which could be WSP, fundamentally affecting the long-term potential benefits of the transition to PEVs. The few studies that do estimate induced generator capacity expansion rely on simplified low-resolution or nonrepresentative load profiles and/or exclude cost-optimized charging or V2G strategies, as summarized in the bottom left cell of Table 1.^27,28–37^ Further details are provided in the Supporting Information.

**Table 1:**
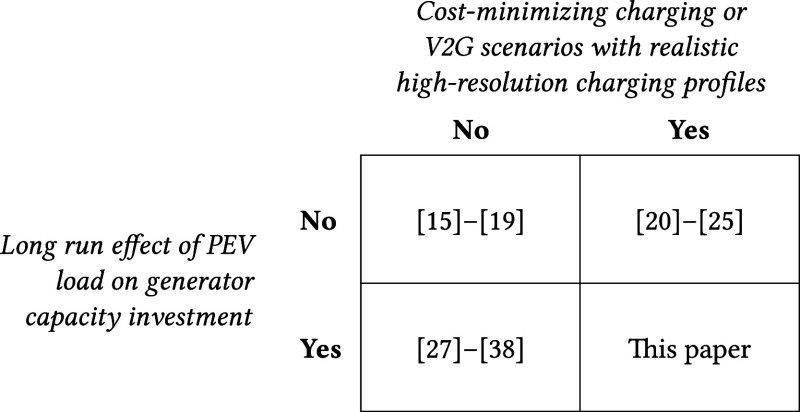
Contribution to the Literature^a^

a We estimate efects of PEV load on power system generator capacity and operations using realistic BEV load profiles, cost-minimizing charging, and V2G scenarios.

We bridge this gap by estimating how power system generation capacity investment incentives are affected by PEV load, cost-minimizing charging, and V2G, addressing the bottom-right corner of Table 1. We use a power system optimization model of PJM—the largest regional grid operator in the U.S., with a generation mix similar to North America’s.^20,21,25,39^ Based on PJM’s interconnection queue and expansion studies, we model the generation fleet across a range of WSP capacity levels. We simulate grid operations both with and without added PEV loads across these WSP capacity levels, and we identify maximum economical WSP capacity levels under each PEV load scenario.

We find that, relative to a reference case with no PEVs, a 10% PEV penetration with uncontrolled PEV charging (immediately after the last trip each day) increases power generation supply primarily from existing fossil fuel plants, consistent with prior studies,^17–21,25^ We estimate that this increases total PJM power grid greenhouse gas and air pollutant emission externalities by $330 per PEV in 2035 (1.6 tons of *CO*_2_ per vehicle or 1% of overall power system externalities), and long run incentives to increase WSP capacity investment are small (300 MW).

In contrast, for cost-minimized charge timing or V2G, long run effects can be substantial: A 10% PEV penetration with cost-minimized charge timing or V2G increases estimated power grid externality estimates by $240 and $610 per PEV (1.0 tons and 2.3 tons of *CO*_2_ *e* per vehicle), respectively, if long-run effects are ignored, but it *reduces* estimated power system externality estimates by $230 and $2200 per PEV, respectively, if long-run effects are modeled by increasing cost-effective levels of WSP generation capacity by 4.3 and 23%.

Our finding that well-timed PEV charging load can trigger infrastructure investment that reduces total power system emissions is consistent with refs 27,35 and our study finds that flexible charge timing and especially V2G can make this effect large enough to flip the direction of PEV charging effects on power system emissions such that increasing PEV charging load results in reduced total grid emissions.

## 2 Materials and Methods

We develop an approach to estimate emission effects of grid intervention technologies in both short-run power system operation and in long-run power system structural changes using a power system operational cost model that has an embedded PEV behavioral module (see workflow of method in Figure 1). The operational model allows a highly resolved representation of real-world PEV operational constraints with options to control charge timing and V2G across hourly, daily and seasonal variations that are not computationally feasible to capture in traditional optimal capacity expansion models,^28–31^ ,^33–37^ and we assess implications for capacity investment by comparing results across a range of capacity investment scenarios to identify the highest profitable capacity investment for each PEV charging profile condition. We then assess changes in greenhouse gas (GHG) emissions and local air pollutants considering the induced capacity expansion. As a case study, we analyze a 2035 version of the PJM Interconnection under three PEV charging conditions: uncontrolled charging, cost-minimizing charging, and vehicle-to-grid (V2G).

**Figure 1:**
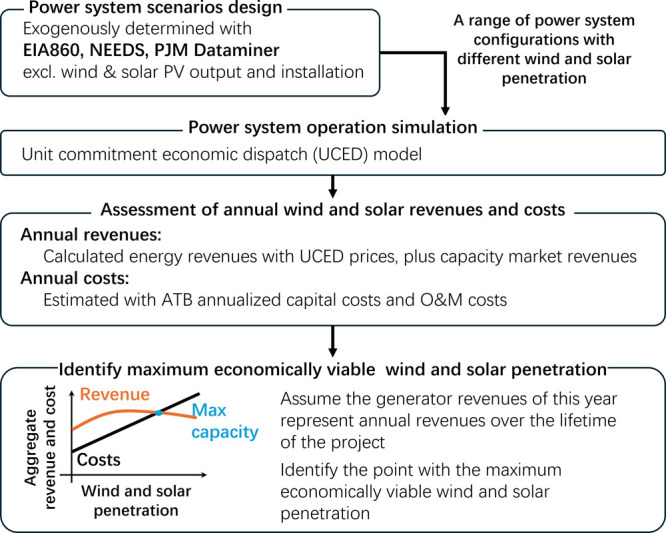
An explanatory diagram of how we translate short-run operational results into long-run capacity investment decisions.

### 2.1 PEV Behavioral Model

We model an electric light-duty vehicle (LDV) fleet that takes up 10% of the LDV fleet in PJM. This PEV stock penetration is plausible, as US PEV penetration in new light-duty vehicle sales reached 10% in 2024, and the US market is expected to grow.^1^ To test result robustness, we also run sensitivity analyses with 20% PEV penetration. As computational power is limited, a simplifying assumption is made that the PEV fleet is made up of generic battery electric vehicles with a 300 mile (480 km) range.

We model three kinds of charging behaviors: uncontrolled charging (UC), cost-minimizing charging (CC), and vehicle-to-grid (V2G). When the electric vehicle charging is uncontrolled, the PEVs engage in convenience charging, meaning PEVs fully charge their battery at the maximum available charging rate at the end of each day at home. Under cost-minimizing charging, when parked the PEVs are assumed to be plugged in and available to be charged dynamically. The charging schedule is co-optimized with the power system economic dispatch during the hours that the PEV is parked and plugged in and within battery capacity constraints. PEVs are required to be fully recharged before the first trip of the following day to be ready for travel. The third charging behavior is V2G. Under V2G, PEVs are always plugged in when parked and can participate in V2G. PEVs that participate in V2G can release energy to or draw energy from the power grid, within operating constraints, allowing charging rates to be negative. The detailed model representation of these operational constraints is described in SI.

In order to represent PEV energy consumption and charging availability across the fleet, we use 15 weighted daily vehicle travel profiles from the National Household Travel Survey (NHTS) to represent 15 groups of PEVs with distinct driving profiles selected to mimic the behavior of the overall fleet as closely as possible, as described in Weis et al. 2014.^28^ These daily driving profiles include the time of the first trip and the last trip of the day, hourly plugged-in availability, and hourly vehicle miles traveled of each hour of the day. The hourly plugged-in availability describes the proportion of PEVs in each PEV behavioral group that are parked and available for charging. Each PEV group has its distinctive driving behavioral pattern. Besides PEVs that are driven, we also model PEVs that are not driven on that day. The NHTS data indicates 30% of vehicles whose owners were surveyed are not active and do not have travel records on the day of survey. Hence, we also include a group of PEVs that do not have driving events for the day. For simplicity and due to data limitations, energy consumption profiles and availability profiles are assumed to be the same every day throughout the year for each group, and driving behavioral patterns are assumed to be homogeneous within each group. The total numbers of PEVs in each region of PJM is calculated as proportional to the region’s population. We compared the 2009 NHTS data set and the 2017 data set and found that daily vehicle miles traveled per household did not change significantly, and thus we adopt the 2009 analysis used in.^28^

### 2.2 Power System Model

We run a unit commitment and economic dispatch (UCED) model to simulate the day-ahead energy market and reserve requirements of the PJM Interconnection. This model was first developed by Lueken et al.^39^ and later adapted by Weis et al.^20,21^ and Bruchon et al.^25^ to incorporate electric vehicle battery charge tracking. The UCED model minimizes variable costs of generators including variable operation and maintenance costs, startup costs, and fuel costs in sliding 48-h optimizing windows. After solving each window, the model accepts the results of the first 24 h and moves forward by 24 h, repeating this process until a full year’s optimization is completed. Our UCED models the PJM Interconnection as 5 transmission-constrained regions, inside each of which there are assumed to be no transmission losses or constraints. In each region, energy demand is constrained to be equal to supply at every time step, and reserve requirements must be met. Operational characteristics of dispatchable generating units, including ramp rates, minimum uptime, and minimum downtime, are modeled through sets of constraints. The operation of solar and wind generators is also modeled, where excessive wind and solar generation can be curtailed when needed.

We incorporate each PEV charging scenario into the UCED model. PEV uncontrolled charging load is added to the baseload, as it is inflexible. PEVs with cost-minimizing charging and V2G are modeled similarly to storage units with limited availability and additional discharge for driving: charging and discharging are limited by the hourly availability of PEVs that are plugged in, and they need to be fully charged before the first trip of the following day. As discussed in methods Section 2.1, 30% of PEVs do not have driving events on any given day. We model this group of PEVs as stationary storage with the same power capacity and energy capacity. During the day, energy depletion from driving is modeled as energy drawn from batteries, realized through extra sets of constraints that track PEV battery states of charge and regulate their charging and discharging rates. The detailed model formulation can be found in the SI.

### 2.3 Power System Scenarios and Data Sources

Given expected wind and solar capacity growth in PJM’s near-term plans and the fact that wind and solar account for most of PJM’s interconnection queue,^40,41^ we model a 2035 PJM power system by increasing wind and solar installed capacity from the current PJM system. We use a PJM system data set compiled by Weis et al. (2016) and updated by Bruchon et al. (2024).^21,25^ It includes generator operational characteristics (e.g., heat rate, capacity, O&M costs, ramp rates, startup cost) from U.S. Energy Information Administration (EIA) Form 860, the 2020 National Electric Energy Data System (NEEDS) data set, and other sources.^42,43^ Generator retirements and additions through 2035 follow reported plans.^25,42^ We assume the same coal power plant capacity as the projected coal capacity in the ‘Accelerated’ scenario of PJM’s 2021 Energy Transition study.^40^ The baseline wind and solar penetration as a percentage of total demand is 22%, based on the ‘Policy’ scenario of the study. Since we analyze beyond the wind and solar penetration of the ‘Policy’ scenario and investigate further decarbonization, we used the ‘Accelerated’ scenario for coal capacity. We first implement all retirement scheduled in Form 860, and retire the remaining coal power plants, from the plants with the oldest NEEDS online years, until the total capacity matches the capacity assumed in the PJM study. The total capacity of dispatchable generators excluding hydro electric generators is 170 GW. This data set models existing storage units based on 2020 EIA Form 860 and storage capacity expansion based on PJM’s energy transition study.^40,42^ Combining pumped hydro storage and battery storage, PJM is assumed to have 5.4 GW (42 GWh) grid-scale storage units. PJM’s DataMiner provides 2020 hly wind and solar generation profiles, load profiles, and transfer limits across PJM.^44^ Fuel prices for each fuel type are retrieved from 2019 EIA Form 923 data.^45^

PJM’s recent energy transition and grid planning study models a “Policy” Scenario where wind and solar supply 22% of total electricity consumption by 2035 with stated policies, and an “Accelerated” Scenario where wind and solar supply 50% of total electricity consumption by 2035.^40^ Active solar PV and wind projects in PJM’s interconnection queue reached 207 and 103 GW, respectively, as of June 2024, indicating mounting interest in wind and solar development.^41^ Based on PJM’s Policy and Accelerated Scenarios and the PJM Interconnection queue, we create 25 high wind and solar penetration scenarios that increase wind and solar generation from 22 to 46% of electricity consumption (excluding PEV and assuming no curtailment) in 1% increments. Solar and wind capacities in our generator fleets range from 24 to 56 GW and 27–66 GW, respectively. To estimate hourly wind and solar generation for each of our 25 scenarios, we combine historic wind and solar output and installed capacity with wind and solar capacity expansion projections from PJM.^40,44^ First, we spatially aggregate 2020 historic observed generation and capacity of solar PV and onshore wind from 20 control zones in PJM to our five regions.^44^ For each of our five regions, we use generation and capacity to calculate hourly capacity factors for solar PV and wind by region. We assume the same capacity factors for onshore and offshore wind given a lack of historic generation data for offshore wind. Offshore wind accounts for up to 15% of wind and solar capacity in our scenarios.

To scale up wind and solar capacity to create each of our 25 scenarios, we assume the capacity ratio of future wind and solar project types will remain the same as projected in PJM’s energy transition study.^40^ First, we use PJM interconnection queue to determine how the capacity expansion is distributed across regions for wind and solar, i.e., what percentage of wind and solar is installed in each of the 5 regions.^44^ The ratio of capacity installation of solar to wind for the whole PJM is determined using the PJM energy transition study,^40^ i.e., what is the ratio of solar capacity installation to wind capacity installation for the whole PJM. With this fixed ratio of wind and solar capacity, we scale up both capacities until total wind and solar generation, calculated as regional capacity times regional capacity factor by generator type, equals the desired combined wind and solar penetration. This method may overestimate future wind or solar capacity factors given that higher resource sites are likely to have already been developed, but efficiency gains in wind and solar technology will at least partly counteract this effect. We test alternative assumptions for the wind-to-solar ratio in Section 3.4.

We run annual UCED simulations for all pairwise combinations of our 25 wind and solar scenarios and 4 PEV charging scenarios (no PEVs and PEVs with uncontrolled charging, cost-minimizing charging, and V2G, described in method Section 2.1), yielding 100 UCED simulations that allow us to quantify PEV impacts at varying wind and solar penetration levels and charging approaches.

### 2.4 Air Emission Externality Costs

We quantify operational stage power system greenhouse gas (GHG) and local air pollutant emissions and estimate consequential air emission externality costs. Emission factors of GHGs and local air pollutants for each generator are retrieved from the National Emissions Inventory (NEI).^46^ For generators that cannot be found in NEI, we substitute with the average emission factor in NEI based on the fuel type. To estimate air emission externality costs, we use a social cost of carbon of $204/ton *CO*_2_ *e* ^47^ for GHGs. For local air pollutants, we assess emissions of sulfur dioxide (SO_2_), nitrogen oxides (NO_X_), ammonia (NH_3_), fine particulate matter (PM_2.5_), and volatile organic compounds (VOCs). Externality costs from local emissions are calculated on a spatially explicit basis using the Air Pollution Emission Experiments and Policy (APEEP) model, version AP3, and mortality risk is monetized using a $8.7 million value of reduced mortality risk.^48^

### 2.5 Assessment of Induced Wind and Solar Capacity Investment

We optimize the UCED model across a range of wind and solar penetration scenarios, identifying the maximum level of wind and solar penetration for which discounted revenue meets or exceeds cost (see Figure 1).

Solar and wind generators have negligible variable costs, and therefore negligible marginal generating costs, in the short-run. However, fixed costs, which equal annualized capital expenditures plus fixed operation and maintenance (O&M) costs, need to be recovered by revenues for wind and solar generators to maintain profitability. To assess the profitability of wind and solar generators, we define net profits as the sum of revenues minus annualized fixed costs. We assume wind and solar generators have two revenue streams: income from the energy market and income from PJM’s capacity market. We assume the 1 year UCED operation that we simulate and optimize repeats throughout generators’ lifetime, as most capacity expansion models do. Hence the revenue-cost balance represents its full lifetime economic viability.

To estimate energy market revenues, we obtain hourly electricity prices for each of five regions from our UCED model. These regional prices are the Lagrange multipliers of each region’s energy balance constraint, which represent the marginal cost of supplying additional load in each region. By summing the product of hourly regional electricity prices and regional generation for wind and for solar, we determine total annual revenues by type in each region from the energy market. To estimate capacity market revenues, we use market mechanisms from PJM’s capacity market, or its Reliability Pricing Model (RPM).^49^ Though the capacity value of wind and solar generators may decrease as penetration increases, it remains uncertain how the capacity market assesses the contribution of wind and solar sources.^7,50^ PJM derates capacity offers from generators based on their effective load carrying capability (ELCC), which reflects generator variability and availability throughout the year. PJM sets the ELCC at 0.15, 0.40, 0.38 for onshore wind, offshore wind and solar PV, respectively.^51^ Wind ELCC is set at 0.233 as onshore wind accounts for one-third of wind capacity expansion. We estimate wind and solar revenues from the capacity market as the product of capacity, the prior ELCC values (assuming the same value for onshore and offshore wind), and the latest average capacity market clearing price (USD 28.92/MW per day).^51^ Regional wind and solar revenues and costs are summed to obtain PJM-wide revenues and costs, which we present in our results.

For computational tractability, we assume system prices, and therefore generator revenues, over this year represent annual prices and revenues over the lifetime of the project, a similar assumption as in capacity expansion models that sample periods from a single year for making investment decisions.

We obtain fixed wind and solar costs from the National Renewable Energy Laboratory’s Annual Technology Baseline (ATB) for year 2030.^52^ As we model year 2035, fixed costs at 2030 would be reasonably representative of fleet average, being the midpoint between the present and the scenario year. Annualized fixed costs are calculated with a real discount rate of 5% and a cost recovery period of 30 years. The data set considers tax incentives in stated policies before the publication of ATB 2022.^52^ Tax credits promulgated by the Inflation Reduction Act of 2022, which have since been largely repealed by the Big Beautiful Bill Act of 2025, were not incorporated in these estimates.

To capture uncertainty in future wind and solar costs, we test the sensitivity of our results to conservative, best-guess, and optimistic ATB cost scenarios, which we refer to as high, mid, and low cost, respectively.^52^

Between the 25 wind and solar capacity levels run for each PEV scenario, we interpolate wind and solar revenues. We find the intersection of fixed costs and revenues and round down the total capacity of wind and solar to the nearest point. And at that point the combined wind and solar capacity installation reaches its maximum profitable level.

### 2.6 Sensitivity Analysis

We run sensitivity analyses to test model sensitivity to our assumptions: (1) exogenous stationary storage capacity; (2) exogenous transmission capacity; (3) fixed wind-to-solar capacity ratio; (4) the size of PEV fleet being 10% of the light-duty vehicle fleet.

To keep the computational burden of this study tractable, stationary storage capacity and transmission capacity are assumed to be fixed. To test sensitivity to the fixed value assumed, we run sensitivity analyses with high transmission capacity and with high stationary storage capacity. In the high transmission scenario, the transmission capacity between each region of PJM is doubled. In the high stationary storage scenario, the capacity of battery storage is expanded from 5.4 GW (46 GWh), to 14 GW (56 GWh), by doubling the capacity of the largest battery storage unit in each region. For regions with pumped hydro storage and without planned battery storage, a 4-h battery storage unit with the power capacity of the largest pumped hydro storage unit is installed. We also test model sensitivity to the fixed wind-to-solar capacity ratio assumption, including a high wind scenario with a wind-to-solar capacity ratio of 1.46 and a high solar scenario with 0.93. Finally, we tested model sensitivity on the size of the PEV fleet. As PEV penetration rises, the benefits of flexible PEV charging and V2G are expected have diminishing returns. We run a high PEV penetration scenario where PEV penetration is doubled from 10% in the base case to 20%.

## 3 Results

We discuss, in turn, results for wind and solar capacity investment, electricity generation, and air emissions induced by PEV charging. We then summarize sensitivity of our results to key assumptions.

### 3.1 Wind and Solar Capacity Investment Induced by PEV Charging

Aggregated results for wind and solar generators are depicted in Figure 2. When aggregated wind and solar revenues equal total fixed costs (labeled intersection points in Figure 2), capacity achieves the maximum level at which capacity can be added profitably. Costs increase with capacity, and we consider low, mid and high cost assumptions. Revenue initially increases with capacity because additional capacity can serve more load, but beyond a critical point revenue begins to decline because additional wind and solar capacity lowers market clearing prices, and the negative effect of lower prices on revenue begins to outweigh the positive effect of higher renewable generation.

**Figure 2:**
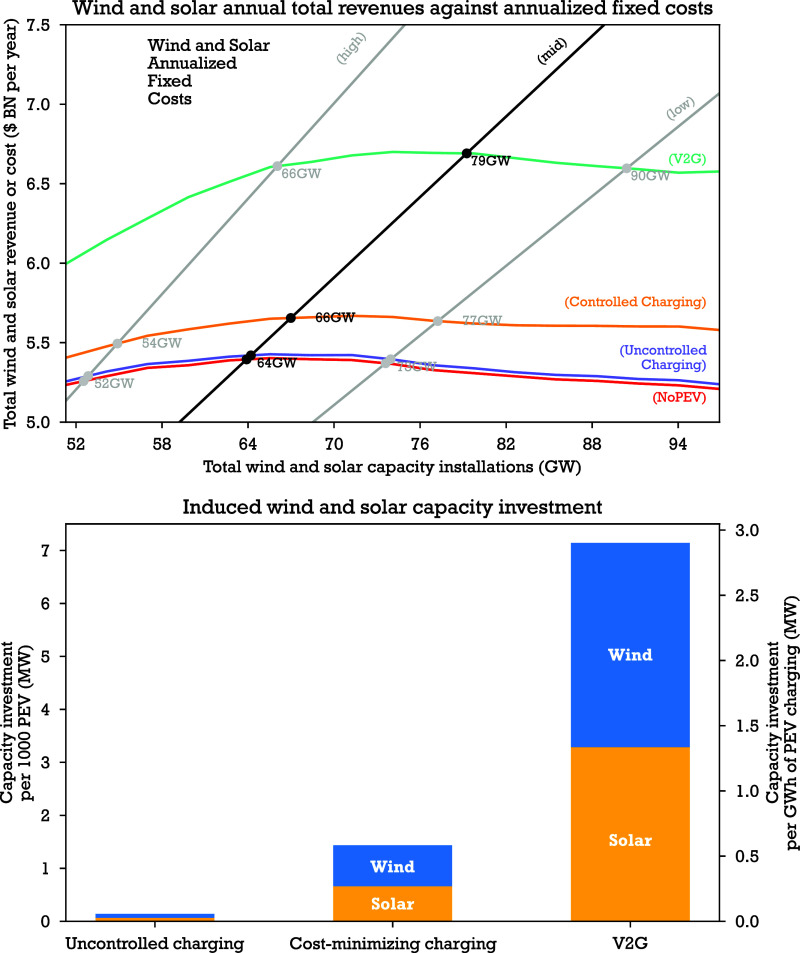
Effect of PEV charging load on the economics of wind and solar capacity investment. *Top panel*: Total revenues and total annualized fixed costs for solar and wind generators, by PEV and PEV charging intervention scenarios and by wind and solar fixed cost scenarios. Labels indicate: (high) conservative fixed cost scenario, (mid) base case fixed cost scenario, (low) optimistic fixed cost scenario. Wind and solar are modeled together at a fixed ratio, as described in Section 2. For each cost case we find the intersection of the annualized total fixed cost curve and the revenue curve, the intersection represents the maximum profitable total wind and solar capacity. Beyond this point additional wind or solar capacity cannot be added profitably. *Bottom panel*: Wind and solar capacity investment induced by PEV charging interventions, including uncontrolled charging (UC), cost-minimizing charging (CC) and vehicle-to-grid (V2G), relative to the NoPEV baseline scenario. Changes of the maximum profitable wind and solar capacity compared to the NoPEV baseline scenario are considered capacity investment induced by PEV and PEV charging interventions. The currency unit is 2024 USD. $ BN is short for billion USD. Further detailed methods are described in method Section 2.3.

In a reference PJM system without PEVs, the estimated maximum profitable level of wind and solar capacity is 63.9 GW (55.9–70.8 GW), assuming moderate (high to low) projected wind and solar fixed cost through 2035. Actually installed wind and solar capacity in PJM as of the end of 2023 totaled 22 GW, with an additional 143 GW in the interconnection queue, indicating ample interest in further wind and solar deployment.^53^

Converting 10% of vehicles to PEVs in PJM can substantially increase the maximum profitable capacity of wind and solar, depending on the charging strategy used. With uncontrolled charging, the maximum profitable capacity of wind and solar increases by only 300 MW, indicating PEV deployment with uncontrolled charging only induces small long-run investment in wind or solar power. But with cost-minimizing charging, the maximum profitable capacity of wind and solar increases by 2.8 GW bringing the total combined wind and solar capacity to 67 GW (a 4.4% increase). With V2G, the maximum profitable wind and solar capacity increases by 15 GW, bringing the total capacity to 80 GW (a 23% increase).

Uncontrolled PEV charging tends to add load over limited periods and when wind and solar generation is low, yielding limited incentives to expand capacity. Conversely, cost-minimizing charging of PEVs allows PEV load to be shifted to periods with low net demand, when wind and solar generation is high, allowing the potential to absorb intermittent renewable generation whenever it occurs. Cost-minimizing charging thus reduces wind and solar curtailment and increases electricity prices in these hours, increasing revenues and making a higher level of wind and solar capacity investment profitable. In addition to charge timing flexibility, V2G also provides storage services, resulting in larger benefits for wind and solar revenue and inducing greater investment.

### 3.2 Electricity Generation Induced by PEV Charging

PEV charging affects power system electricity generation directly by increasing demand or, in the case of V2G, providing electricity storage to the grid, and it also affects generation indirectly through induced investment in wind and solar capacity (Figure 3). Here, we separate these two factors by comparing two sets of scenarios that (1) ignore or (2) include induced wind and solar investment by PEVs. Within each set of scenarios, we quantify the effect of PEVs on electricity generation by comparing scenarios with PEVs and different charging strategies to a reference scenario without PEVs.

**Figure 3:**
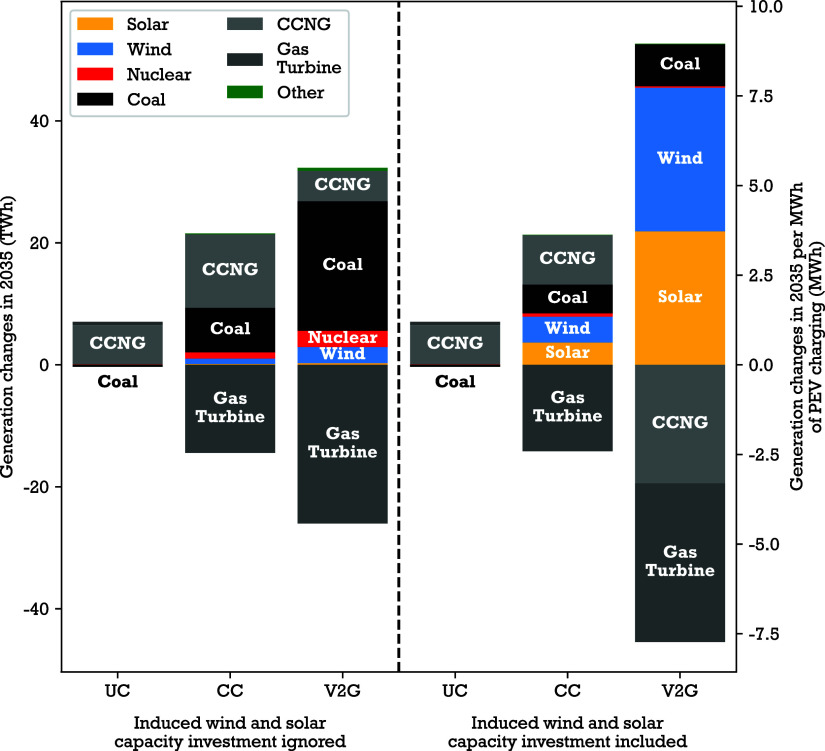
Effect of PEV charging load on power generation. Changes in annual generation by fuel type, relative to the NoPEV case, for each PEV charging scenario when ignoring versus including induced wind and solar capacity investment. When accounting for induced wind and solar investment, Wind and solar capacity and therefore generation vary across PEV charging scenarios. ‘UC’: uncontrolled charging; ‘CC’: cost-minimizing charging; ‘V2G’: vehicle-to-grid. ‘CCNG’ fuel type includes combined cycle natural gas generators. ‘Other’ fuel types include biomass, fossil waste, fuel cell, hydro, landfill gas, municipal solid waste, nonfossil waste, and oil or gas steam.

#### 3.2.1 Ignoring Long-Run Capacity Investment Effects

When we ignore induced wind and solar capacity investment, PEVs with uncontrolled charging increase combined cycle natural gas generation by 6.4 TWh relative to the NoPEV case, or 1.1 MWh per MWh of PEV charging (higher than 1 due to rounding and losses during charging). Uncontrolled charging demand roughly coincides with daily load peaks, when combined cycle natural gas generators tend to operate on the margin. Wind and solar power experience minimal curtailment (0.5% of total generation) in the no-PEV reference scenario, and the timing of uncontrolled charging does not align with curtailed renewables, so PEV load does not mitigate curtailment.

Cost-minimizing charging and V2G provide additional flexibility of demand timing, allowing the system to use otherwise curtailed renewables. But due to low wind and solar curtailment rates, cost-minimizing charging only increases wind and solar generation by 0.18 MWh per MWh of PEV charging, and the larger effect of shifting charge timing is an increase low-marginal-cost coal generation by 1.2 MWh, combined cycle natural gas generation by 2.0 MWh, and nuclear generation by 0.17 MWh per MWh of PEV charging. Demand flexibility enabled by cost-minimizing charging also leads to a 2.5 MWh reduction in high-marginal-cost gas turbine natural gas generation per MWh of PEV charging. Similar changes occur with V2G charging. V2G only increases wind and solar generation by 0.50 MWh and nuclear generation by 0.46 MWh per MWh of PEV charging, while increasing coal generation by 3.6 MWh, combined cycle natural gas generation by 0.84 MWh. Gas turbine natural gas generation is reduced by 4.4 MWh per MWh of PEV charging.

#### 3.2.2 Including Long-Run Capacity Investment Effects

When we account for induced wind and solar capacity expansion, the effect of PEVs on the generation mix changes substantially under cost-minimizing charging and V2G scenarios but not under the uncontrolled charging scenario. Uncontrolled charging does not induce significant wind and solar capacity investment (0.2 GW, Figure 2), so generation mix changes are nearly the same when accounting for or ignoring induced wind and solar capacity investment. But cost-minimizing charging and V2G scenarios induce larger wind and solar investment, resulting in increasing wind and solar generation that displaces coal- and gas-fired generation. With cost-minimizing charging, additional flexibility increases wind and solar generation by 1.3 MWh per MWh of PEV charging, coal generation by 0.80 MWh, and combined cycle natural gas generation by 1.4 MWh. Gas turbine generation is reduced by 2.4 MWh per MWh of PEV charging. With V2G, additional flexibility increases wind and solar generation by 7.7 MWh per MWh of PEV charging, and coal generation by 1.2 MWh. Gas turbine generation is reduced by 4.4 MWh, and combined cycle natural gas by 3.3 MWh per MWh of PEV charging.

As a result of generation mix changes, total system costs differ under different charging scenarios. Uncontrolled charging adds 1.8% to system costs regardless of whether induced wind and solar capacity is ignored or included. For V2G, however, when ignoring induced wind and solar capacity investment, total system costs are 6% lower than without PEVs (a $400 per PEV per year reduction). When accounting for induced wind and solar capacity investment, total system costs under V2G are 13% lower than without PEVs (an $880 per PEV per year reduction).

### 3.3 Air Emission Externalities Induced by PEV Charging

PEV charging affects power system greenhouse gas (GHG) and local air pollutant emissions through its effect on induced capacity investment and electricity generation (Figure 4). We estimate externality costs of these emissions using a $204 per mt CO_2_eq social cost of carbon^47^ and using the AP3 model for estimating air pollution-related mortality risk with a $8.7 M value of reduced mortality risk.^48^

**Figure 4:**
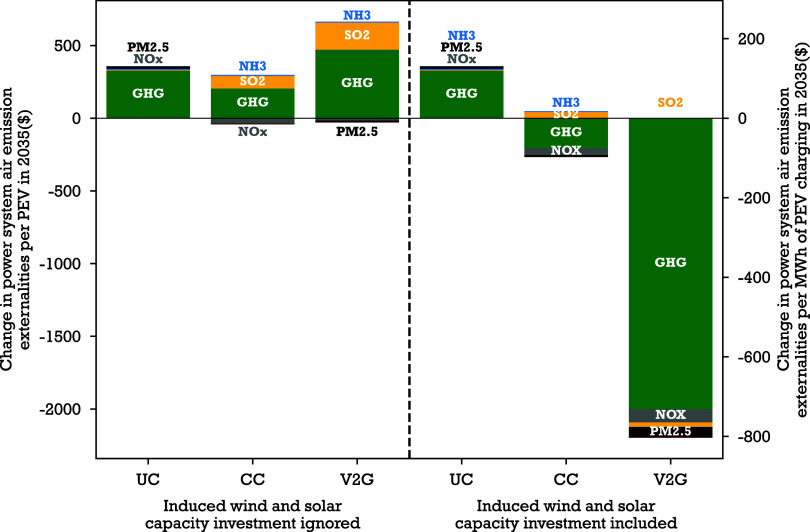
Effect of PEV charging on power system air emission externalities. Change in total power system air emission externalities per PEV and per MWh of PEV charging in 2035, relative to the NoPEV scenario, under each PEV charging scenario when ignoring versus including induced wind and solar capacity investment. The ‘ignored’ scenarios use generation portfolios given by PJM’s grid planning study for 2035.^40^ The ‘included’ scenarios consider wind and solar capacity at maximum profitable capacity, as described in Section 2. ‘GHG’ is short for greenhouse gases. When induced wind and solar capacity investment is ignored, PEV load increases power system air emission externalities. When it is included, uncontrolled PEV load increases power system air emission externalities, but cost-minimizing charging (CC) and vehicle-to-grid (V2G) scenarios induce enough wind and solar capacity investment to produce a net reduction in power system air emission externalities.

When we ignore induced wind and solar capacity investment, PEV charging increases emissions externalities by increasing fossil-fuel generation (Figure 3). As shown in Figure 4, PEV adoption increases overall 2035 power system air emission externalities by $330, $240 or $610 per PEV when charging is uncontrolled, cost-minimizing, or V2G, respectively.

When we account for induced wind and solar capacity investment, uncontrolled PEV charging still increases power system air emission externalities by $330 per PEV, as before, but because cost-minimizing charging and V2G induce new wind and solar capacity investment, PEV charging *reduces* total power system air emission externalities by $230 or $2200 per PEV for cost-minimizing charging and V2G, respectively.

### 3.4 Sensitivity Analysis

Under all sensitivity scenarios, V2G benefits persist in (1) inducing wind and solar capacity investment; (2) reducing system cost; and (3) reducing power system emission externalities (as summarized in Table 2). However, substantial variations exist depending on the tested parameters. Detailed results for each sensitivity scenario are described in the SI, and we summarize the core findings below.

**Table 2:** Sensitivity Analysis: Robustness of V2G Results to Parameter Variations^a^

input parameters	baseline value	tested value	induced wind and solar capacity investment per PEV	annual system cost reduction per PEV per year	annual emissions externality reduction per PEV	interpretation
stationary storage capacity	5.4 GW	14 GW	–74%	–80%	–71%	PEV V2G has smaller (but positive) benefits for power systems with more stationary storage.
	42 GWh	56 GWh				
transmission capacity	varies	2× baseline	+21%	+25%	+32%	PEV V2G has larger benefits in power systems with more transmission capacity.
PEV fleet penetration	10%	20%	–15%	–19%	–32%	Larger PEV fleets yield diminishing returns.
wind to solar capacity ratio	1.17	1.43	+4%	+14%	+4%	PEV V2G benefits vary with assumed wind-to-solar ratio.
		0.96	+20%	+14%	+4.5%	

a Estimates are relative to the base case V2G effect estimates.

Changing from the base case to the high renewable fixed cost scenario led to a 10% decrease in induced investment in wind and solar capacity per PEV, a 3.6% decrease in system cost reduction per PEV, and a 15% decrease in emission externality reduction per PEV. Changing from the base case to the low renewable fixed cost scenario led to a 9.9% increase in induced investment in wind and solar capacity per PEV, a 0.22% decrease in system cost reduction per PEV, and a 15% decrease in emission externality reduction per PEV. Though fixed costs vary substantially across the three scenarios, V2G contribution to system cost reductions and emission externality reductions are substantial, indicating the robustness of results against fixed cost levels. Meanwhile, since the real discount rate assumed by the model also determines fixed cost annualization, the fixed cost sensitivity results also indicate the robustness of results against real discount rate.

Increasing stationary storage capacity from 5.4 GW (42 GWh) to 14 GW (56 GWh) led to a 74% decrease in induced investment in wind and solar capacity per PEV, an 80% decrease in system cost reduction per PEV, and a 71% decrease in emission externality reduction per PEV. The significant dilution of the V2G value in a power system with high stationary storage capacity suggests the substitution of stationary storage by V2G.

Doubling transmission capacity led to a 21% increase in induced investment in wind and solar capacity per PEV, a 25% increase in system cost reduction per PEV, and a 32% increase in emission externality reduction per PEV. The significant enhancement of V2G value in a power system with high transmission capacity suggests synergies between transmission capacity expansion and V2G.

Increasing PEV fleet penetration from 10 to 20% resulted in a 15% decrease in induced investment in wind and solar capacity per PEV, a 19% decrease in system cost reduction per PEV, and a 32% decrease in emission externality reduction per PEV. Despite doubling PEV penetration, the contribution per PEV to cost savings and emission reductions was not severely diluted, highlighting the scalability of PEV integration.

Adjusting the wind-to-solar capacity ratio from 1.17 to 1.43 led to a 4% increase in induced investment in wind and solar capacity per PEV, a 14% increase in system cost reduction per PEV, and a 4% increase in emission externality reduction per PEV. Adjusting the wind-to-solar capacity ratio from 1.17 to 0.96 led to a 20% increase in induced investment in wind and solar capacity per PEV, a 14% increase in system cost reduction per PEV, and a 4.5% increase in emission externality reduction per PEV. The higher performance of V2G under the high solar scenario could be attributed to the relatively low costs of solar PV compared to wind.

The sensitivity analysis shows variations in system cost reduction and emission externality reduction across different parameter settings. However, the value of V2G remains positive in all cases, demonstrating its potential to deliver consistent benefits across a variety of system conditions.

## 4 Discussion and Limitations

### 4.1 Limitations and Caveats

Our estimates are based on optimal grid operations and optimal use of PEV charging flexibility. In practice, benefits may be somewhat lower because of limitations in the ability to forecast load, variable renewable generation, and PEV availability, and to coordinate charging decisions among millions of PEV households.

We acknowledge that V2G may potentially change battery degradation, and device/infrastructure upgrades could be required for V2G. If such costs are accounted for during V2G operation, there may be fewer energy arbitrage opportunities for V2G. As a result, V2G could produce lower cost reductions and emission reduction benefits. However, some studies have suggested Li-ion batteries are very durable to cycling, and additional battery cycling from V2G may not be problematic.^54–60^

We treat wind and solar together assuming a fixed 54–46% capacity ratio based on the mix of wind and solar resources in the interconnection queue. Moreover, we assume future wind and solar capacity expansion will follow the current geographical distribution. Actual wind and solar build-outs may be more strategic, adding uncertainty to our estimates. For example, optimal wind and solar development may result in less curtailment than under a fixed ratio assumption. Hence flexible loads and storage provided by cost-minimizing charging and V2G would render less valuable. We run sensitivity analyses with high wind capacity and high solar capacity, respectively. In the high wind case, V2G’s cost reduction is 14% higher than under the base case, and the emission externality reduction is 4.0% higher. In the high solar case, V2G’s cost reduction is 14% higher than under the base case, and the emission externality reduction is 4.5% higher. The analysis reveals variability in the magnitude of results; however, the value of V2G remains positive and substantial under all tested conditions, reinforcing its potential to deliver economic and environmental benefits. More details are provided in the SI.

Our estimated benefits are based on wind and solar investment decisions driven by economic viability. Other factors, such as national, state, and local regulations, incentives, process delays, or political factors may also influence realized investment decisions in practice. For example, wind and solar PV projects make up 93% of PJM’s interconnection queue, but a growing backlog of new wind and solar project requests indicates the interest in investment is not matched by the actual build-out.^61^ Furthermore, we analyze a state of long-run equilibrium, where all profitable wind and solar generators are built, representing long-run perfect competition where free entry and exit result in implementation of all profitable construction (in contrast to single firm profit maximization, where marginal cost equals marginal revenue). In doing so we ignore the time lag between market, investment, and the actual buildout, and we ignore other deviations from perfect competition outcomes. For simplicity, data availability, and computational reasons, we also assume that generation profiles and expansion costs are identical across all wind and across all solar generators, regardless of location. Location-resolved values could improve upon our estimates.

Our power system model accounts for planned capacity changes in the generator fleet covered in EIA Form 860 and projected in PJM’s 2021 Energy Transition study, but we do not consider the potential impacts of future policies on the power system.^40^ As a result, coal-burning generation capacity in this study may differ from the real world, depending on the policy environment. We assume the same coal power plant capacity to match the projected coal capacity in the ‘Accelerated’ scenario of PJM’s 2021 Energy Transition study.^40^ However, state and federal regulations such as the Revised Clean Air Act Section 111­(d) could eliminate coal power plants by 2035, and other changes in policy could accelerate or delay coal retirement.

We do not model transmission capacity expansion endogenously, but we do run a sensitivity analysis with double the transmission capacity of the base case to investigate this uncertainty. In the high transmission cases, V2G yields 25% more system cost reduction and 32% more emission reduction compared with the base case. This indicates synergies instead of competition exist between transmission capacity expansion and V2G. More details are provided in the SI.

We only model flexible loads from PEVs and none from other sources. Other flexible loads and increases in PEV fleet size may dilute the benefits of PEV cost-minimizing charging and V2G. We run a sensitivity analysis where 8.4 GW stationary storage is added to the system and a case with a PEV penetration of 20% instead of 10% in the base case. In the high storage case, the cost reduction and emission externality reduction benefits per PEV are reduced by 80 and 71%. In operation, V2G provides energy storage services, filling the same role as utility-scale storage units. In the high PEV penetration case, the cost reduction and emission externality reduction benefits per PEV are only reduced by 19 and 32%, respectively. More details are provided in the SI.

We note that alternative “uncontrolled” charging schedules, such as daytime charging rather than charging after the last trip of the day, could potentially create different incentives for wind and solar capacity investment, which could also affect consequential emissions of PEV charging without cost-minimizing charging.^27^

Our analysis is specific to the PJM region, but preliminary findings of working studies in other regions suggest that flexible load timing can reduce long-run emissions more broadly.^62,63^

### 4.2 Discussion

We find that a 10% increase in PJM PEV adoption induces only minor additional wind and solar capacity investment when each vehicle is charged (uncontrolled) after the last trip of the day. But when PEV charge timing is scheduled to minimize cost, adding PEV load can substantially increase profitable levels of wind and solar capacity investment so much that net power system air emissions externality costs actually drop. When PEVs have bidirectional V2G capabilities, the drop in net air emissions externality costs is much higher.

Our estimates suggest that the adoption of cost-minimizing charging or V2G reduces the power system air emission externality cost consequences of PEV adoption by $230 and $2200 per PEV per year, respectively, in 2035 PJM, suggesting a policy rationale for incentivizing adoption of cost-minimizing charging and V2G. Our estimates also suggest that consequential life cycle air emissions externalities of PEV adoption may be smaller than estimated in prior studies^25^ if the PEVs use cost-minimizing charging or V2G.

Home chargers capable of receiving signals from a utility and adjusting the timing and rate of charging in response are needed to enable cost-minimizing charging. For V2G, this term sometimes implies bidirectional flow to and from the power grid, which requires special grid connections typically unavailable at the household level. But the term V2G sometimes implies only bidirectional flow from the vehicle to the home to displace household load without requiring a bidirectional connection between the home and the power grid (also known as vehicle-to-home). Our analysis is agnostic to these V2G variations so long as household load exceeds vehicle discharge in the analysis. However, the V2G cases that we model implicitly assume bidirectional communication between the vehicle and the power grid, which is needed to determine whether PEVs are plugged in and how much headroom PEV batteries have to charge or discharge at a given moment. The development of such communication systems and algorithms to aggregate information and distribute control would be necessary to realize large V2G adoption of the type modeled here.

We use the term “long-run consequential emissions” for our estimates and avoid the term “long-run marginal emissions” used in ref 26 because marginal emissions are a special case of consequential emissions for which the change in question is a single unit (for discrete quantities or the derivative for continuous quantities).^64^ Our study involves a 10% change in PEV adoption, which is too large to be considered marginal.

We focus on PEV charging load, but other kinds of grid interventions with flexible loads also have the potential to induce wind and solar capacity investment. For example, building electrification with the deployment of air source heat pumps can increase grid emissions due to additional load;^65–67^ however, combined with load control, the additional load can incentivize solar and wind investment, which serves not only the heat pump load but also the rest of the power system, thus helping to facilitate the decarbonization of the whole power system. To harness the discussed benefits, it is suggested that power system operators and energy management service providers actively explore both the technical and economic viability and market designs for potential grid flexibility solutions. With mature business models, these grid flexibility solutions may help the power system reduce both costs and emissions in its transition to carbon neutrality.

## Supplementary Material



## Notes

Code and data availability, sensitivity analysis, detailed methods, Figures S1–S19, and Tables S1–S7 (PDF)

## References

[1] Alsauskas, O. ; Connelly, E. ; Daou, A. ; Gouy, A. ; Huismans, M. ; Le Marois, J.-B. ; McDonagh, S. ; Petropoulos, A. ; Teter, J. ; Jenness, E. ; Copier, J. J. ; Lombardo, T. ; O’Riordan, V. ; Sery, J. ; Global EV outlook 2024 moving towards increased affordability, Apr. 2024. [Online]. Available: https://iea.blob.core.windows.net/assets/a9e3544b-0b12-4e15-b407-65f5c8ce1b5f/GlobalEVOutlook2024.pdf.

[2] Archsmith J., Muehlegger E., Rapson D. S. (2022). Future paths of electric vehicle
adoption in the united states: Predictable determinants, obstacles,
and opportunities. Environmental and Energy
Policy and the Economy.

[3] Gagnon, P. ; Brown, M. ; Steinberg, D. ; Brown, P. ; Awara, S. ; Carag, V. ; Cohen, S. ; Cole, W. ; Ho, J. ; Inskeep, S. , 2022 Standard Scenarios Report: A US Electricity Sector Outlook, National Renewable Energy Lab.(NREL): Golden, CO (United States), 2022.

[4] Mitigation pathways compatible with 1.5°c in the context of sustainable development, In Global Warming of 1.5°C: IPCC Special Report on Impacts of Global Warming of 1.5°C above Pre-industrial Levels in Context of Strengthening Response to Climate Change, Sustainable Development, and Efforts to Eradicate Poverty, Intergovernmental Panel on Climate Change (IPCC) , Ed., Cambridge: Cambridge University Press, 2022; pp 93–174, doi: 10.1017/9781009157940.004. https://www.cambridge.org/core/books/global-warming-of-15c/mitigation-pathways-compatible-with-15c-in-the-context-of-sustainable-development/051AC891C0952E62DEF2510593BC1C10 isbn: 978–1-009–15795–7. [Online]. Available: (visited on 07/25/2023).

[5] Wiser, R. H. ; Mills, A. ; Seel, J. ; Levin, T. ; Botterud, A. , Impacts of Variable Renewable Energy on Bulk Power System Assets, Pricing, and Costs, Lawrence Berkeley National Lab. (LBNL): Berkeley, CA (United States), Nov. 29, 2017. doi: 10.2172/1411668. https://www.osti.gov/biblio/1411668

[6] Chen X., Zhang H., Xu Z., Nielsen C. P., McElroy M. B., Lv J. (2018). Impacts of fleet types and charging
modes for electric vehicles on
emissions under different penetrations of wind power. Nature Energy.

[7] Mills A. D., Levin T., Wiser R., Seel J., Botterud A. (2020). Impacts of
variable renewable energy on wholesale markets and generating assets
in the united states: A review of expectations and evidence. Renew. Sustainable Energy Rev..

[8] Das S., Hittinger E., Williams E. (2020). Learning is not enough: Diminishing
marginal revenues and increasing abatement costs of wind and solar. Renewable Energy.

[9] Craig M. T., Jaramillo P., Hodge B.-M., Williams N. J., Severnini E. (2018). A retrospective
analysis of the market price response to distributed photovoltaic
generation in california. Energy Policy.

[10] Bird, L. ; Cochran, J. ; Wang, X. , Wind and Solar Energy Curtailment: Experience and Practices in the United States,” National Renewable Energy Lab. (NREL): Golden, CO (United States), 2014.

[11] Jones, L. E. Renewable Energy Integration: Practical Management of Variability, Uncertainty, and Flexibility in Power Grids. Academic Press, 2017. https://www.google.com/books?hl=zh-CN&lr=&id=b_6pDQAAQBAJ&oi=fnd&pg=PP1&dq=Flexibility+in+21st+Century+Power+Systems&ots=uFNkIQB0EG&sig=P1KbKXppuWZM4KnJyX-sjKy07QM

[12] Kroposki B., Johnson B., Zhang Y., Gevorgian V., Denholm P., Hodge B.-M., Hannegan B. (2017). Achieving
a 100% renewable
grid: Operating electric power systems with extremely high levels
of variable renewable energy. IEEE Power and
Energy Magazine.

[13] Impram S., Nese S. V., Oral B. (2020). Challenges of renewable
energy penetration
on power system flexibility: A survey. Energy
Strategy Rev..

[14] Mai T., Denholm P., Brown P., Cole W., Hale E., Lamers P., Murphy C., Ruth M., Sergi B., Steinberg D., Baldwin S. F. (2022). Getting to 100%: Six strategies for
the challenging last 10%. Joule.

[15] Shi R., Li S., Zhang P., Lee K. Y. (2020). Integration of renewable energy sources
and electric vehicles in v2g network with adjustable robust optimization. Renewable Energy.

[16] Chen J., Wang F., He X., Liang X., Huang J., Zhang S., Wu Y. (2022). Emission mitigation
potential from
coordinated charging schemes for future private electric vehicles. Appl. Energy.

[17] Holland S. P., Mansur E. T., Muller N. Z., Yates A. J. (2016). Are there environmental
benefits from driving electric vehicles? the importance of local factors. American Economic Review.

[18] Holland S. P., Mansur E. T., Muller N. Z., Yates A. J. (2020). Decompositions and
policy consequences of an extraordinary decline in air pollution from
electricity generation. American Economic Journal:
Economic Policy.

[19] Holland S. P., Kotchen M. J., Mansur E. T., Yates A. J. (2022). Why marginal CO2
emissions are not decreasing for US electricity: Estimates and implications
for climate policy. Proc. Natl. Acad. Sci. U.
S. A..

[20] Weis A., Michalek J. J., Jaramillo P., Lueken R. (2015). Emissions and cost
implications of controlled electric vehicle charging in the u.s. PJM
interconnection. Environ. Sci. Technol..

[21] Weis A., Jaramillo P., Michalek J. (2016). Consequential life cycle air emissions
externalities for plug-in electric vehicles in the PJM interconnection. Environ. Res. Lett..

[22] Forrest K. E., Tarroja B., Zhang L., Shaffer B., Samuelsen S. (2016). Charging a
renewable future: The impact of electric vehicle charging intelligence
on energy storage requirements to meet renewable portfolio standards. J. Power Sources.

[23] Tarroja B., Zhang L., Wifvat V., Shaffer B., Samuelsen S. (2016). Assessing
the stationary energy storage equivalency of vehicle-to-grid charging
battery electric vehicles. Energy.

[24] Nunes P., Brito M. C. (2017). Displacing natural
gas with electric vehicles for grid
stabilization. Energy.

[25] Bruchon M., Chen Z. L., Michalek J. (2024). Cleaning up
while changing gears:
The role of battery design, fossil fuel power plants, and vehicle
policy for reducing emissions in the transition to electric vehicles. Environ. Sci. Technol..

[26] Gagnon P. J., Bistline J. E. T., Alexander M. H., Cole W. J. (2022). Shortrun marginal
emission rates omit important impacts of electric-sector interventions. Proc. Natl. Acad. Sci. U. S. A..

[27] Holland, S. P. ; Mansur, E. T. ; Yates, A. ; Decarbonization and Electrification in the Long Run, Rochester, NY, May 1, 2022. [Online]. Available: https://papers.ssrn.com/abstract=4122811

[100] Hanig L., Harper C. D., Nock D., Michalek J. J. (2025). Driving the grid
forward: How electric vehicle adoption
shapes power system infrastructure and emissions. Proc. Natl. Acad. Sci. U.S.A..

[28] Weis A., Jaramillo P., Michalek J. (2014). Estimating the potential of controlled
plug-in hybrid electric vehicle charging to reduce operational and
capacity expansion costs for electric power systems with high wind
penetration. Applied Energy.

[29] Nopmongcol U., Grant J., Knipping E., Alexander M., Schurhoff R., Young D., Jung J., Shah T., Yarwood G. (2017). Air quality impacts of electrifying
vehicles and equipment
across the united states. Environ. Sci. Technol..

[30] Carrión M., Domínguez R., Zárate-Miñano R. (2019). Influence
of the controllability of electric vehicles on generation and storage
capacity expansion decisions. Energy.

[31] Brown T., Schlachtberger D., Kies A., Schramm S., Greiner M. (2018). Synergies
of sector coupling and transmission reinforcement in a costoptimised,
highly renewable european energy system. Energy.

[32] Sheppard C. J. R., Bauer G. S., Gerke B. F., Greenblatt J. B., Jenn A. T., Gopal A. R. (2019). Joint optimization
scheme for the
planning and operations of shared autonomous electric vehicle fleets
serving mobility on demand. Transport. Res.
Record.

[33] Manríquez F., Sauma E., Aguado J., de la Torre S., Contreras J. (2020). The impact of electric vehicle charging
schemes in
power system expansion planning. Appl. Energy.

[34] Sheppard C. J. R., Jenn A. T., Greenblatt J. B., Bauer G. S., Gerke B. F. (2021). Private
versus shared, automated electric vehicles for u.s. personal mobility:
Energy use, greenhouse gas emissions, grid integration, and cost impacts. Environ. Sci. Technol..

[35] Gagnon P., Cole W. (2022). Planning for the evolution of the electric grid with a long-run marginal
emission rate. iScience.

[36] Owens J., Miller I., Geṇcer E. (2022). Can vehicle-to-grid
facilitate the
transition to low carbon energy systems?. Energy
Advances.

[37] Jenn A. (2023). Emissions
of electric vehicles in california’s transition to carbon neutrality. Appl. Energy.

[39] Lueken R., Apt J. (2014). The Effects of Bulk
Electricity Storage on the PJM Market. Energy
Syst..

[40] PJM , Energy Transition in PJM: Frameworks for Analysis, Dec. 15, 2021. [Online]. Available: https://pjm.com/-/media/committees-groups/committees/mrc/2021/20211215/20211215-item-09-energy-transition-in-pjm-whitepaper.ashx.

[41] PJM State of the Market—2024 , Monitoring Analytics, Jun. 7, 2024. [Online]. Available: https://www.monitoringanalytics.com/reports/PJM_State_of_the_Market/2024.shtml

[42] Form EIA-923 Detailed Data with Previous form Data (EIA-906/920)—US Energy Information Administration (EIA) (Jun. 7, 2024), [Online]. Available: https://www.eia.gov/electricity/data/eia923/

[43] National electric energy data system (NEEDS) . (Apr. 23, 2023), [Online]. Available: https://www.epa.gov/power-sector-modeling/national-electric-energy-data-system-needs

[44] Data miner 2. (Jun. 7, 2024), [Online]. Available: https://dataminer2.pjm.com/

[45] “Form EIA-860 detailed data with previous form data (EIA-860a/860b).” (Jun. 7, 2024), [Online]. Available: https://www.eia.gov/electricity/data/eia860/

[46] OAR, US EPA . “National emissions inventory (NEI).” (Jun. 2, 2015), Available: https://www.epa.gov/air-emissions-inventories/national-emissions-inventory-nei

[47] EPA Report on the Social Cost of Greenhouse Gases: Estimates Incorporating Recent Scientific Advances, Nov. 2023.

[48] Clay K., Jha A., Muller N., Walsh R. (2019). External costs
of transporting petroleum
products: Evidence from shipments of crude oil from north dakota by
pipelines and rail. Energy J..

[49] PJM , PJM Manual 18: PJM Capacity Market, Feb. 9, 2023.

[50] Bhagwat P. C., Iychettira K. K., Richstein J. C., Chappin E. J. L., De
Vries L. J. (2017). The effectiveness of capacity markets in the presence
of a high portfolio share of renewable energy sources. Util. Policy.

[51] 2023/2024 BRA Effective Load Carrying Capability (ELCC) Class Ratings, 2021. [Online]. Available: https://www.pjm.com/-/media/planning/res-adeq/elcc/elcc-class-ratings-for-2023-2024-bra.ashx.

[52] Vimmerstedt, L. ; Stehly, T. ; Akar, S. ; Sekar, A. ; Mirletz, B. ; Stright, D. ; Augustine, C. ; Beiter, P. ; Bhaskar, P. ; Blair, N. ; Cohen, S. ; Cole, W. ; Duffy, P. ; Feldman, D. ; Gagnon, P. ; Kurup, P. ; Murphy, C. ; Ramasamy, V. ; Robins, J. ; Zuboy, J. ; Oladosu, D. ; Hoffmann, J. , 2022 Annual Technology Baseline (ATB) Cost and Performance Data for Electricity Generation Technologies, DOE Open Energy Data Initiative (OEDI); National Renewable Energy Laboratory (NREL), 5716, 2022. 10.25984/1871952.

[53] PJM—Serial Service Request Status (Jun. 11, 2024), [Online]. Available: https://www.pjm.com/planning/service-requests/serial-service-request-status

[54] Gasper P., Saxon A., Shi Y., Endler E., Smith K., Thakkar F. M. (2023). Degradation and
modeling of large-format commercial
lithium-ion cells as a function of chemistry, design, and aging conditions. J. Energy Storage.

[55] Chowdhury N. R., Smith A. J., Frenander K., Mikheenkova A., Lindströom R.
W., Thiringer T. (2024). Influence
of state of charge window
on the degradation of tesla lithium-ion battery cells. J. Energy Storage.

[56] Leippi A., Fleschutz M., Davis K., Klingler A.-L., Murphy M. D. (2024). Optimizing
electric vehicle fleet integration in industrial demand response:
Maximizing vehicle-to-grid benefits while compensating vehicle owners
for battery degradation. Appl. Energy.

[57] Sagaria S., van der Kam M., Boström T. (2025). Vehicle-to-grid impact on battery
degradation and estimation of v2g economic compensation. Appl. Energy.

[58] Gong J., Wasylowski D., Figgener J., Bihn S., Rücker F., Ringbeck F., Sauer D. U. (2024). Quantifying the impact of v2x operation
on electric vehicle battery degradation: An experimental evaluation. eTransportation.

[59] Naumann, M. ;, “Techno-economic evaluation of stationary battery energy storage systems with special consideration of aging,” Ph.D. dissertation, Technische Universität München, 2018. [Online]. Available: https://mediatum.ub.tum.de/1434981

[60] Naumann M., Spingler F. B., Jossen A. (2020). Analysis and
modeling of cycle aging
of a commercial LiFePO4/graphite cell. J. Power
Sources.

[61] Silverman, A. ; Wendling, Z. ; Rizal, K. ; Outlook for pending generation in the PJM interconnection queue, May 8, 2024. [Online]. Available: https://www.energypolicy.columbia.edu/publications/outlook-for-pending-generation-in-the-pjm-interconnection-queue/.

[62] Sambasivam, P. D. B. ; Bhaskar, A. ; Kockelman, P. K. M. ; Greer, D. ; Leibowicz, P. D. B. D. ; Economic and Environmental Impacts of Electric Vehicle Smart-Charging Programs on the u.s. Power Sector, 2023. https://www.semanticscholar.org/paper/ECONOMIC-AND-ENVIRONMENTAL-IMPACTS-OF-ELECTRIC-ON-Sambasivam-Bhaskar/d13aabce16e1152fe5de8bf6ceb5cdad862ed9a0

[63] Bhandarkar, R. ; Luo, Q. ; Dimanchev, E. ; Jenkins, J. D. ;, Are EVs Cleaner than We Think? Evaluating Consequential Greenhouse Gas Emissions from EV Charging, Apr. 24, 2025. Available: http://arxiv.org/abs/2504.17632

[64] Current Methods for Life Cycle Analyses of Low-Carbon Transportation Fuels in the United States, in collab. with Committee on Current Methods for Life Cycle Analyses of Low-Carbon Transportation Fuels in the United States, Board on Environmental Studies and Toxicology, Board on Agriculture and Natural Resources, Board on Energy and Environmental Systems, Division on Earth and Life Studies, Division on Engineering and Physical Sciences, and National Academies of Sciences, Engineering, and Medicine. National Academies Press: Washington, DC, Oct. 26, 2022, doi: 10.17226/26402. isbn: 978-0-309-27393-0. [Online]. Available: https://www.nap.edu/catalog/26402

[65] Dai B., Qi H., Dou W., Liu S., Zhong D., Yang H., Nian V., Hao Y. (2020). Life cycle energy, emissions and
cost evaluation of CO2 air source heat pump system to replace traditional
heating methods for residential heating in china: System configurations. Energy Convers. Manage..

[66] Liu S., Li Z., Dai B., Zhong Z., Li H., Song M., Sun Z. (2019). Energetic,
economic and environmental analysis of air source transcritical
CO2 heat pump system for residential heating in china. Applied Thermal Engineering.

[67] Carroll P., Chesser M., Lyons P. (2020). Air source
heat pumps field studies:
A systematic literature review. Renew. Sustainable
Energy Rev..

