# Supplementary material for: Negative Electric Vehicle Emissions: Vehicle-to-Grid Can Incentivize Enough Wind and Solar Investment to Reverse EV Charging Emissions
Source: Environ Sci Technol. 2025 Sep 27;59(39):21090–101. doi: 10.1021/acs.est.5c06944 (PMC12509313; doi:10.1021/acs.est.5c06944)
Supplement: Supplementary file 1 [file es5c06944_si_001.pdf]

Supplementary Information for 'Negative Electric Vehicle Emissions: Vehicle-to-Grid Can Incentivize Enough Wind and Solar Investment to Reverse EV Charging Emissions'

Jiahui Chen[a,b], Michael T. Craig[a,c], Jeremy Michalek **[b,d,e], Matthew Bruchon[b], Parth Vaishnav[a]

[a]*School for Environment and Sustainability, University of Michigan, Ann Arbor, MI 48109, USA*

[b]*Department of Engineering and Public Policy, Carnegie Mellon University, Pittsburgh, PA 15213, USA*

[c]*Department of Industrial and Operations Engineering, University of Michigan, Ann Arbor, MI 48109, USA*

[d]*Department of Mechanical Engineering, Carnegie Mellon University, Pittsburgh, PA 15213, USA*

[e]*Department of Civil and Environmental Engineering, Carnegie Mellon University, Pittsburgh, PA 15213, USA*

Summary: 32 pages, 19 figures, 7 table

Email address: jmichalek@cmu.edu (Jeremy Michalek **)

Brief Description of Supporting Information

Supplementary Information for 'Negative Electric Vehicle Emissions: Vehicle-to-Grid Can Incentivize Enough Wind and Solar Investment to Reverse EV Charging Emissions'

Supporting Information includes:
- 32 pages
- 19 figures (Figures S1–S19)
- 7 tables (Tables S1–S7)

Brief Description: Code and data availability, sensitivity analysis, detailed methods, Figures S1–S19, Tables S1–S7.

S1. Data and Code Availability

Data and code used in this research are stored on a public repository (https://github.com/jia-hui-chen0/Long-term-emission-effects-of-grid-intervention.git)

S2. Supplementary Literature Review

This section provides supplementary literature review to the main body (S1). Current studies assess emission impacts of plug-in electric vehicle adoption and charging interventions mainly with two approaches, focusing either on the power system operation or on the power system capacity expansion. The first approach assesses emission impacts induced by grid intervention with a fixed power system[1]–[6]. Tu et al. 2020 and Holland et al. 2022 used marginal emission factors to evaluate emission impacts of PEV charging intervention.[1], [2] Chen et al. 2018 used a power system dispatch model to assess the emission impacts of charging intervention in fixed power systems [7]. However, Owens et al. and Gagnon et al. [8], [9] note that, in the long run, grid interventions like EV adoption can induce changes in the trajectory of power system generator capacity expansion, and this structural change of the power system also has climate implications.

Other relevant studies employ capacity expansion models (CEMs). However, CEMs typically do not model granular short-term power system constraints, and many of these studies make simplification assumptions about grid operation [8], [10], [11]. Brown et al. 2018 assessed cost reduction effects of grid flexibility options provided by the building sector and the transportation sector [10]. Though their model included PEV with smart charging and V2G, it represented PEV flexibility with stationary storage of 50% capacity of PEVs. This simplification can be necessary for CEMs but lacks representation of operational constraints that are vital to understanding PEV flexibility. Weis et al. 2014 managed to join the force of an operational cost model and a capacity expansion model[12]. The study ran power system simulation for 4 representative weeks of a year and used the results as inputs for a capacity expansion model. As a result, the study's scalability was limited. Moreover, the study did not model V2G. Owens et al. 2022, based on a CEM, represented V2G operation in the model with detailed operational constraints, informed by real-world travel records [8]. However, they only allow V2G to participate in the ancillary service market but not in the energy market, which limits the potential of V2G. Moreover, the study only considers a hypothetical optimal power system that is built from scratch and does not consider effects of installed fossil fuel generators on grid interventions, which, can increase system emissions with large scale deployment of energy storage and[13] While the investigation done by existing studies have been insightful and fruitful, a holistic view that combines power system operation and capacity expansion helps answer the question better, as it is the short-term operational constraints that ultimately determines the system operation outcome.

Furthermore, by ignoring transmission constraints between grid regions, along with the possibility that generation and EV loads are added in different regions, the study omits probable spatial mismatch between V2G and generation. As for the spatial nuances, V2G service is best available in load centers [14], [15], whereas much of variable renewable energy generation capacity is located in distant and rural areas [16], [17]. This mismatch of the spatial distribution of demand and supply also affects the value of grid flexibility options such as flexible PEV load.

Table S1: Comparison of method and materials of existing studies and this study. VRE is short for variable renewable energy.

Author and year	Modeling of operational constraints of grid intervention	Realistic BEV charging profiles	Endogenous capacity expansion	Consideration of existing fossil fuel generators	Analysis of impacts of grid intervention on VRE deployment	Endogenous power system operation modeling	V2G modeling
Owens et al. 2022 [8]	✓		✓		✓	✓	✓
Holland et al. 2022 [1]				✓			
Gagnon & Cole 2022 [9]			✓	✓	✓	✓	
Carrión et al. 2015 [18]	✓		✓	✓	✓		
Shi et al. 2020 [5]	✓			✓		✓	✓
Manríquez et al. 2020 [11]	✓			✓		✓	✓
Jenn et al. 2020 [19]		✓	✓	✓		✓	
Weis et al. 2014 [12]	✓	✓	✓		✓	✓	
Weis et al. 2015 [20]	✓	✓		✓	✓	✓	
Nunes & Brito 2017 [21]	✓	✓				✓	✓
Forrest et al. 2016 [22]	✓	✓		✓		✓	✓
Tarroja et al. 2016 [23]	✓	✓				✓	✓
This study	✓	✓	✓	✓	✓	✓	✓

S3. Sensitivity analysis

S3.1. Higher stationary storage

In the base case, the PJM power system has 5.4 GW(42 GWh) grid-scale storage units. The assumed stationary storage is constant against varying wind and solar power penetration levels. To test the robustness of our results against the addition of more stationary storage capacity, we run the analysis with more battery storage capacity, where the total stationary storage capacity is increased to 14 GW (56 GWh).

Shown in Figure S1, V2G increases the maximum profitable capacity of wind and solar capacity by 4.2 GW (a 6% increase), significantly lower than under the base case: 15 GW (from 64 GW to 79 GW, a 23% increase). As the system's demand for grid flexibility is saturated by the addition of stationary storage, benefits provided by V2G significantly diminish.

As a result of lower induced wind and solar capacity investment, the wind and solar generation changes induced by V2G under the high battery scenario are also smaller than the base case (shown in Figure S2). When induced capacity investment is considered, wind and solar generation increases (15 TWh) are much lower than under the base case (45 TWh).

Due to changes of generation mix, total system costs differ under different charging scenarios. When ignoring induced wind and solar capacity investment, total system costs under V2G are 0.20% higher than without PEVs ($13 per PEV per year increase). When accounting for induced wind and solar capacity investment, total system costs under V2G are 3% lower than without PEVs ($180 per PEV per year reduction). System cost reduction per PEV per year is 80% lower than the base case.

As a result of changes discussed above, V2G's emission externality reduction benefits are also significantly diminished (see Figure S3). When considering induced wind and solar capacity investment, V2G reduces air emission externalities by $630 per PEV, compared with $2200 under the base case.

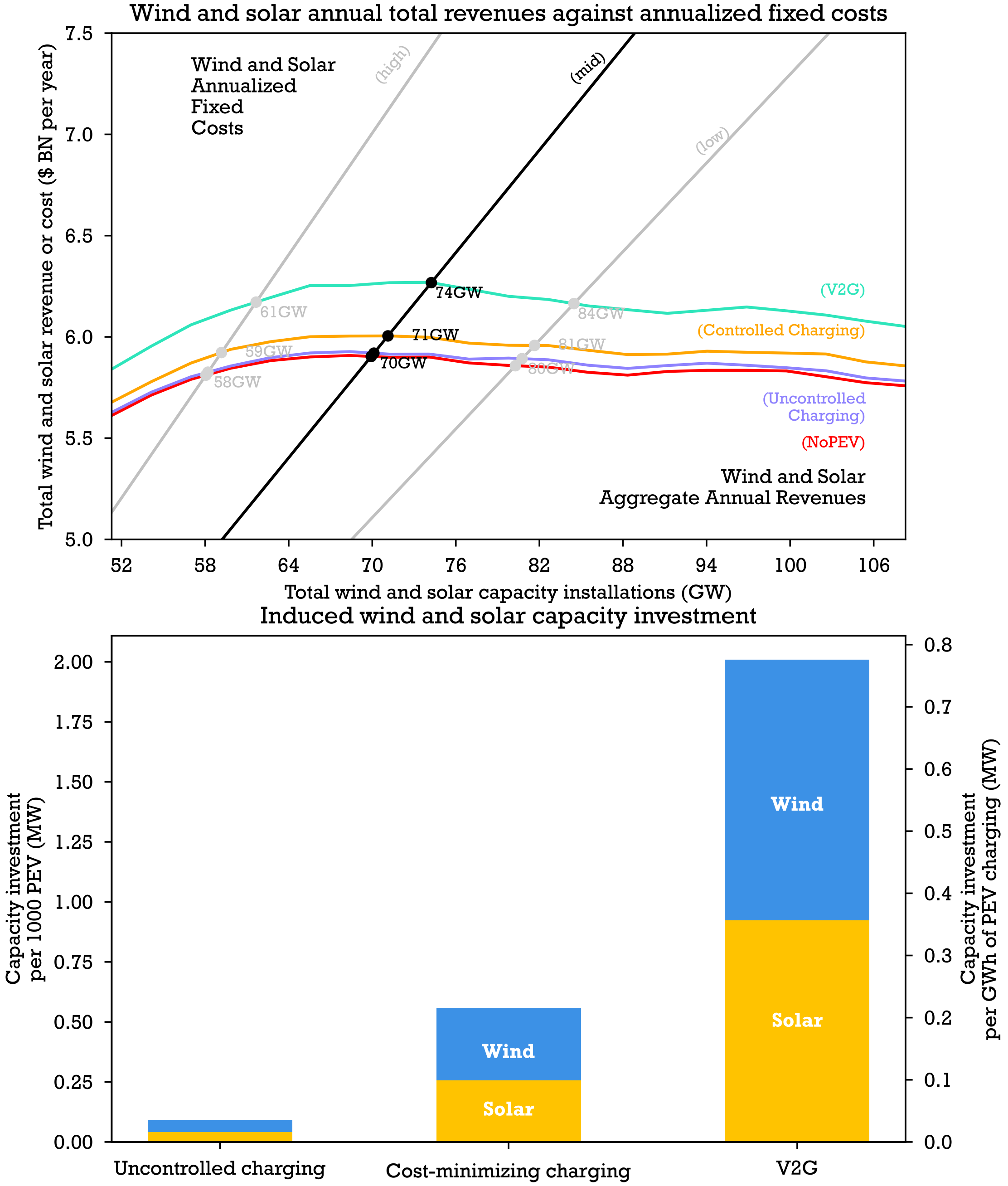

Figure S1: Effect of PEV charging load on the economics of wind and solar capacity investment. Top panel: Total revenues and total annualized fixed costs for solar and wind generators, by PEV and PEV charging intervention scenarios and by wind and solar fixed cost scenarios. **All results in this figure are run with additional stationary storage than the base case (14 GW compared with 5.4 GW).** All other parameters are the same. Note: (high): conservative fixed cost scenario, (mid): base case fixed cost scenario, (low): optimistic fixed cost scenario. Bottom panel: Wind and solar capacity investment induced by PEV charging interventions, including uncontrolled charging (UC), cost-minimizing charging (CC) and vehicle-to-grid (V2G), relative to the NoPEV baseline scenario. The currency unit is 2024 USD. $ BN is short for billion USD. Further detailed methods are described in the method section.

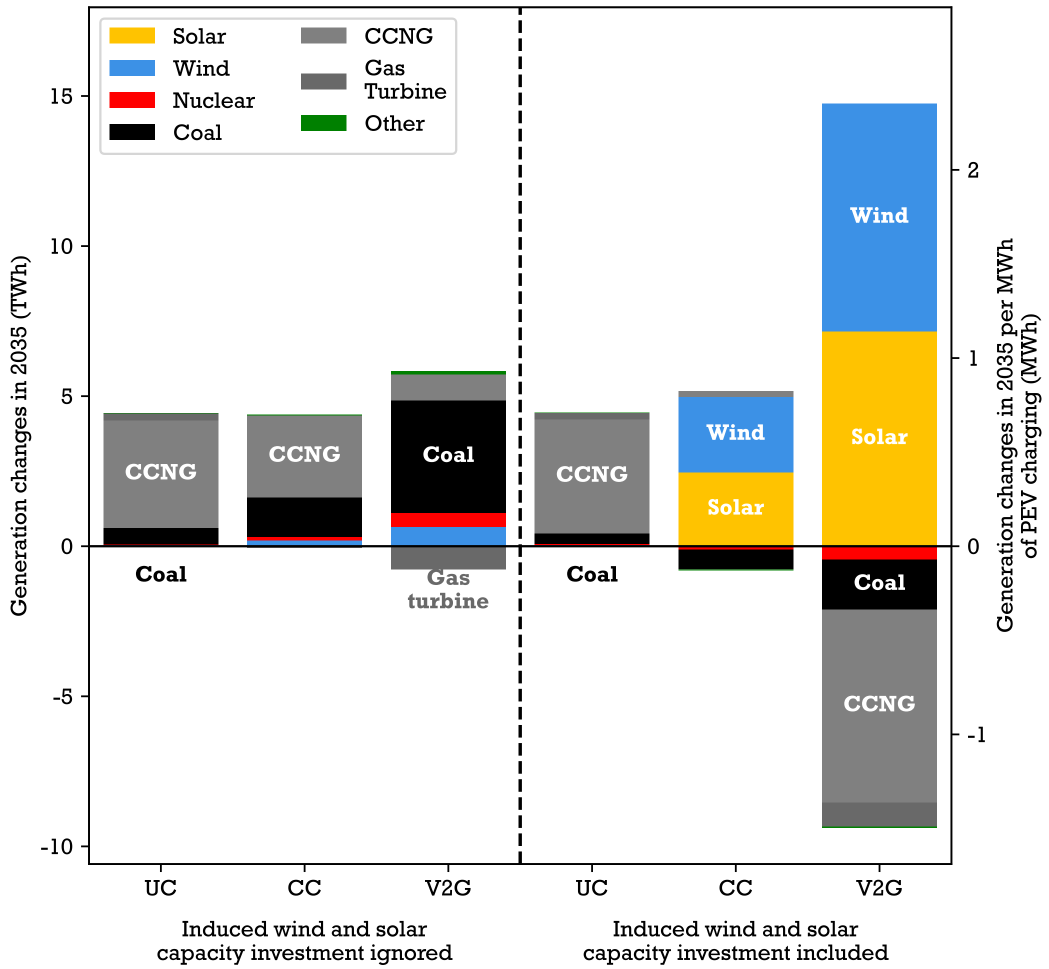

Figure S2: Effect of PEV charging load on power generation. Changes in annual generation by fuel type, relative to the NoPEV case, for each PEV charging scenario when ignoring versus including induced wind and solar capacity investment. **All results in this figure are run with additional stationary storage than the base case (14 GW compared with 5.4 GW in the base case).** All other parameters are the same. 'CCNG' fuel type includes combined cycle natural gas generators. 'Other' fuel types include biomass, fossil waste, fuel cell, hydro, landfill gas, municipal solid waste, non-fossil waste, and oil or gas steam.

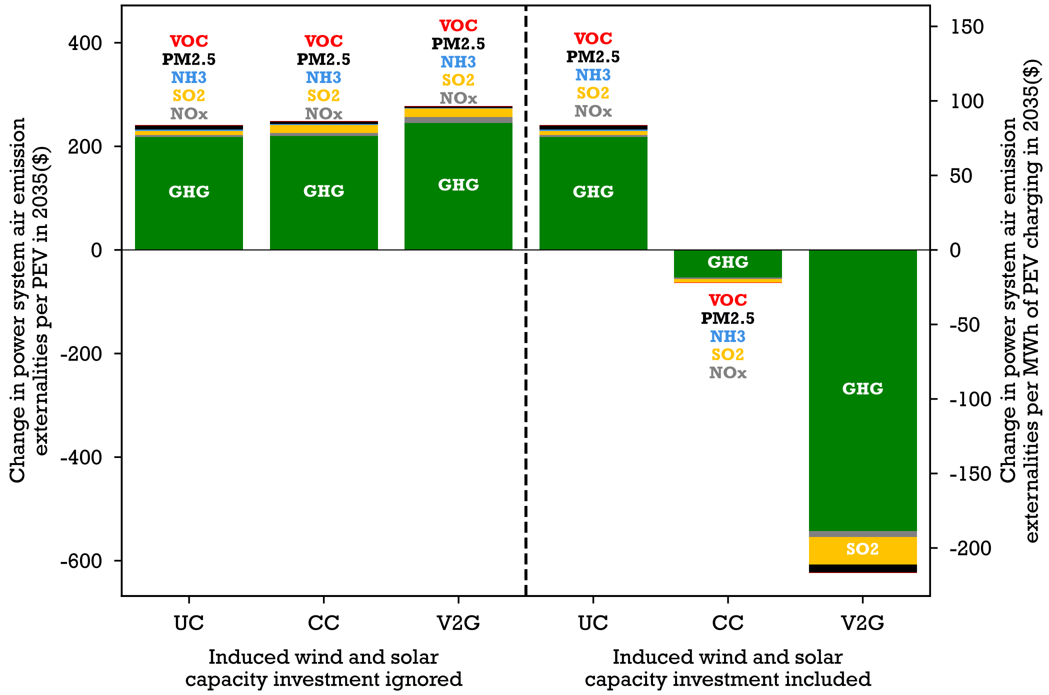

Figure S3: Effect of PEV charging on power system air emission externalities. Change in total power system air emission externalities per PEV and per MWh of PEV charging in 2035, relative to the NoPEV scenario, under each PEV charging scenario when ignoring versus including induced wind and solar capacity investment. **All results in this figure are run with additional stationary storage than the base case (14 GW compared with 5.4 GW in the base case).** The 'ignored' scenarios use generation portfolios given by PJM's grid planning study for 2035 [24]. The 'included' scenarios consider wind and solar capacity at maximum profitable capacity, as described in Method and Materials.

S3.2. Higher transmission

To test the robustness of our results against the addition of more transmission capacity, we run the analysis with double the transmission capacity compared to the base case.

Shown in Figure S4, V2G increases the maximum profitable capacity of wind and solar capacity by 20 GW (from 71 GW to 91 GW, a 28% increase), higher than under the base case: 15 GW (from 64 GW to 79 GW, a 23% increase). V2G benefits from better interconnectedness with the transmission capacity expansion.

As a result of more induced wind and solar capacity investment, the wind and solar generation changes induced by V2G under the high transmission scenario are also larger than the base case (shown in Figure S5). When induced capacity investment is considered, wind and solar generation increases (55 TWh) are 22% higher than under the base case (45 TWh).

When ignoring induced wind and solar capacity investment, total system costs under V2G are 5.2% lower than without PEVs ($340 per PEV per year reduction). When accounting for induced wind and solar capacity investment, total system costs under V2G are 18% lower than without PEVs ($1100 per PEV per year reduction). System cost reduction per PEV per year is 25% higher than the base case.

As a result of changes discussed above, V2G reduces air emission externalities by $2900 per PEV, 32% higher compared with $2200 under the base case (see Figure S6).

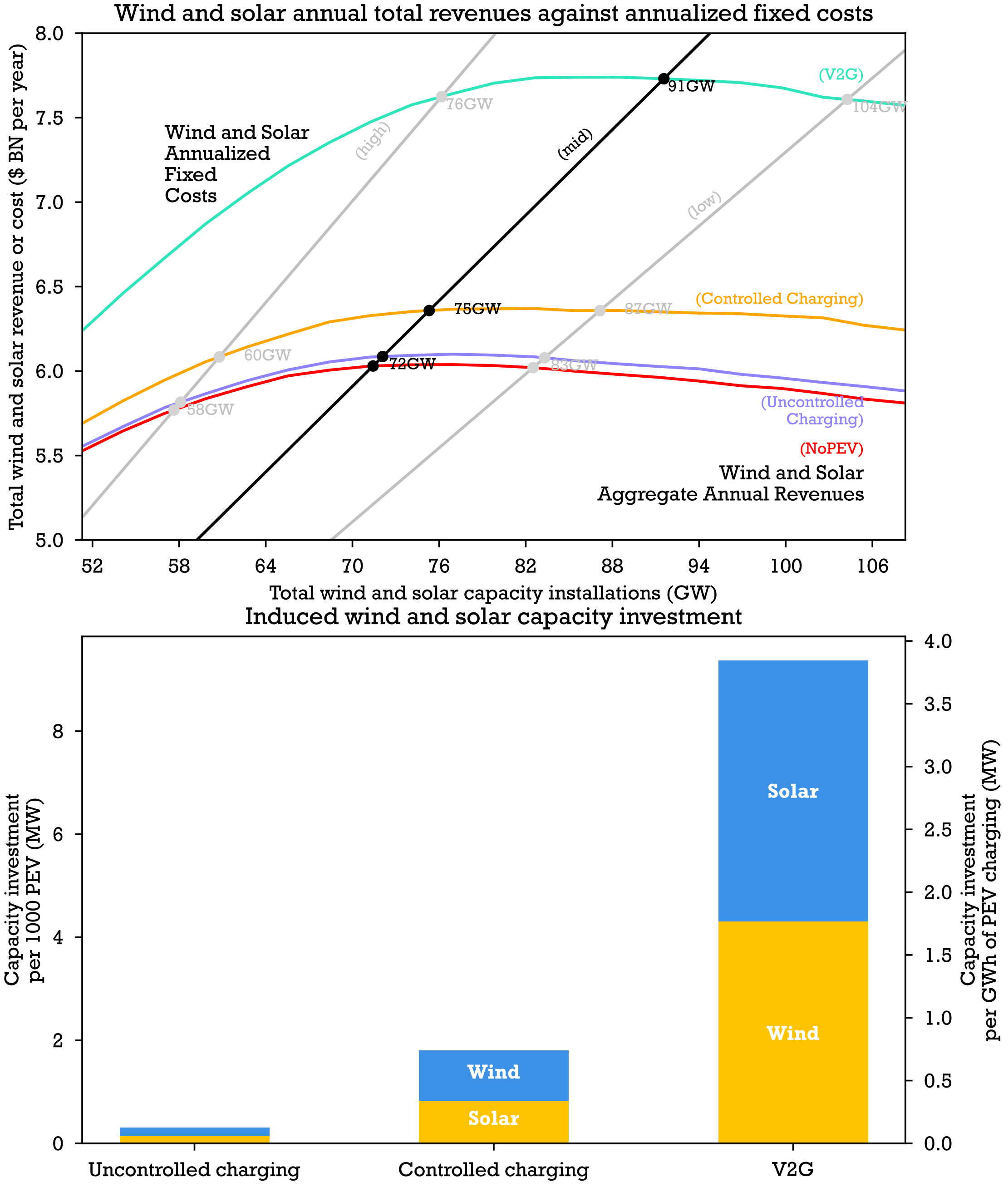

Figure S4: Effect of PEV charging load on the economics of wind and solar capacity investment. Top panel: Total revenues and total annualized fixed costs for solar and wind generators, by PEV and PEV charging intervention scenarios and by wind and solar fixed cost scenarios. **All results in this figure are run with twice as much transmission capacity as the base case.** All other parameters are the same. Note: (high): conservative fixed cost scenario, (mid): base case fixed cost scenario, (low): optimistic fixed cost scenario. Bottom panel: Wind and solar capacity investment induced by PEV charging interventions, including uncontrolled charging (UC), cost-minimizing charging (CC) and vehicle-to-grid (V2G), relative to the NoPEV baseline scenario. The currency unit is 2024 USD. $ BN is short for billion USD. Further detailed methods are described in the method section.

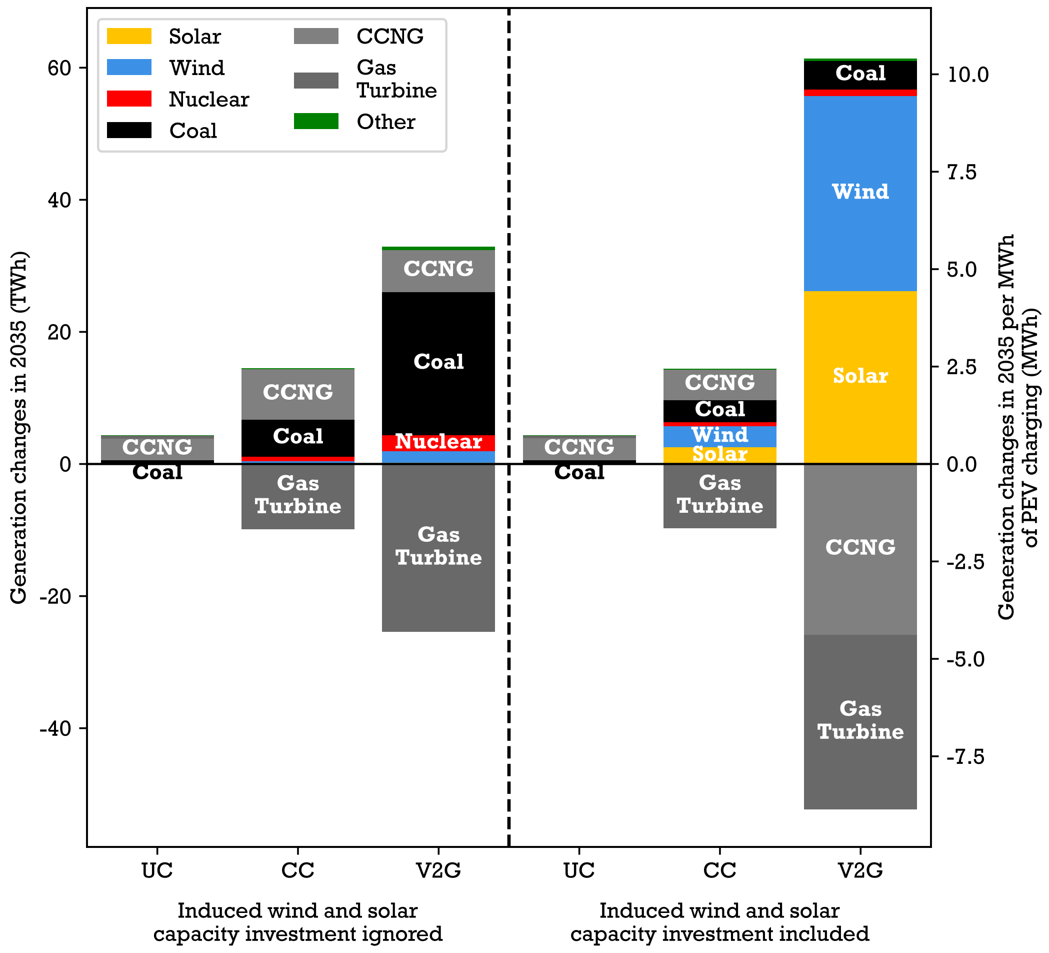

Figure S5: Effect of PEV charging load on power generation. Changes in annual generation by fuel type, relative to the NoPEV case, for each PEV charging scenario when ignoring versus including induced wind and solar capacity investment. **All results in this figure are run with twice as much transmission capacity as the base case.** All other parameters are the same. 'CCNG' fuel type includes combined cycle natural gas generators. 'Other' fuel types include biomass, fossil waste, fuel cell, hydro, landfill gas, municipal solid waste, non-fossil waste, and oil or gas steam.

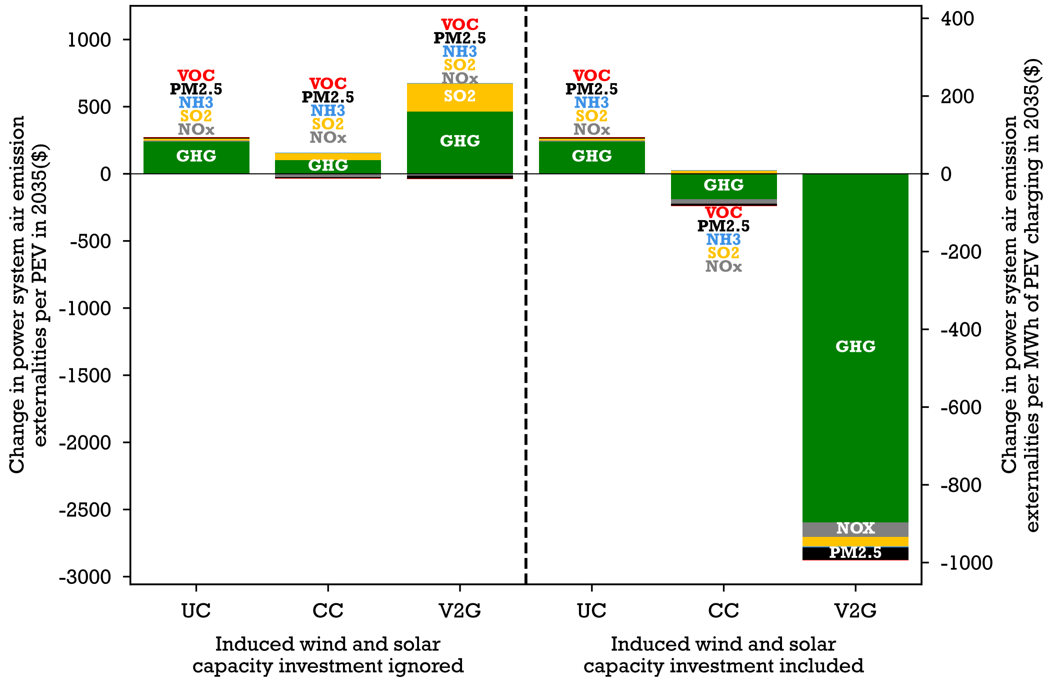

Figure S6: Effect of PEV charging on power system air emission externalities. Change in total power system air emission externalities per PEV and per MWh of PEV charging in 2035, relative to the NoPEV scenario, under each PEV charging scenario when ignoring versus including induced wind and solar capacity investment. **All results in this figure are run with twice as much transmission capacity as the base case.** The 'ignored' scenarios use generation portfolios given by PJM's grid planning study for 2035 [24]. The 'included' scenarios consider wind and solar capacity at maximum profitable capacity, as described in Method and Materials.

S3.3. Higher PEV penetration

In the base case, we assume the PEV fleet accounts for 10% of PJM's light-duty passenger vehicles. To test the robustness of our results with more PEVs in the system, we run the analysis with more PEVs, where the number of PEVs is doubled.

Shown in Figure S7, with a larger PEV fleet, the total wind and solar capacity investment induced by V2G increases significantly. Under a high PEV penetration scenario, V2G increases the maximum profitable capacity of wind and solar capacity by 24 GW (a 39% increase) for a combined wind and solar capacity of 64 GW with no PEVs. The increases are 60% higher than under the base case: 15 GW (a 23% increase) increase for a combined capacity with no PEVs of 64 GW. On the per GWh charging demand basis, however, the induced capacity investments with higher PEV penetration (2.3 MW/GWh of PEV charging) are lower than the base case (2.9 MW/GWh of PEV charging). The capacity investment induction benefits of V2G are only diluted by 20% despite doubling the number of PEVs.

As a result of higher induced wind and solar capacity investment, the wind and solar generation changes induced by V2G under the high battery scenario are also higher than the base case (shown in Figure S8). When induced capacity investment is considered, wind and solar generation increases (68 TWh) are 51% higher than under the base case (45 TWh). On the per MWh of PEV charging basis, wind and solar generation increases by 5.8 MWh for every MWh of PEV charging, compared with 7.8 MWh under the base case. The wind and solar generation increase is diluted by 26% due to the addition of PEVs to the system.

When ignoring induced wind and solar capacity investment, total system costs under V2G are 5.2% lower than without PEVs ($170 per PEV per year reduction). When accounting for induced wind and solar capacity investment, total system costs under V2G are 19% lower than without PEVs ($620 per PEV per year reduction). As a result of higher wind and solar installed capacity and generation discussed above, doubling the number of PEVs also increases V2G's air emission externality reduction benefits (shown in Figure S9). With a PEV penetration of 20%, total air emission externalities are reduced by $1500 per PEV per year, 32% less compared with the base case. Though doubling the number of PEVs dilutes emission externality reduction on the per PEV basis, the benefit dilution lags behind the penetration increase.

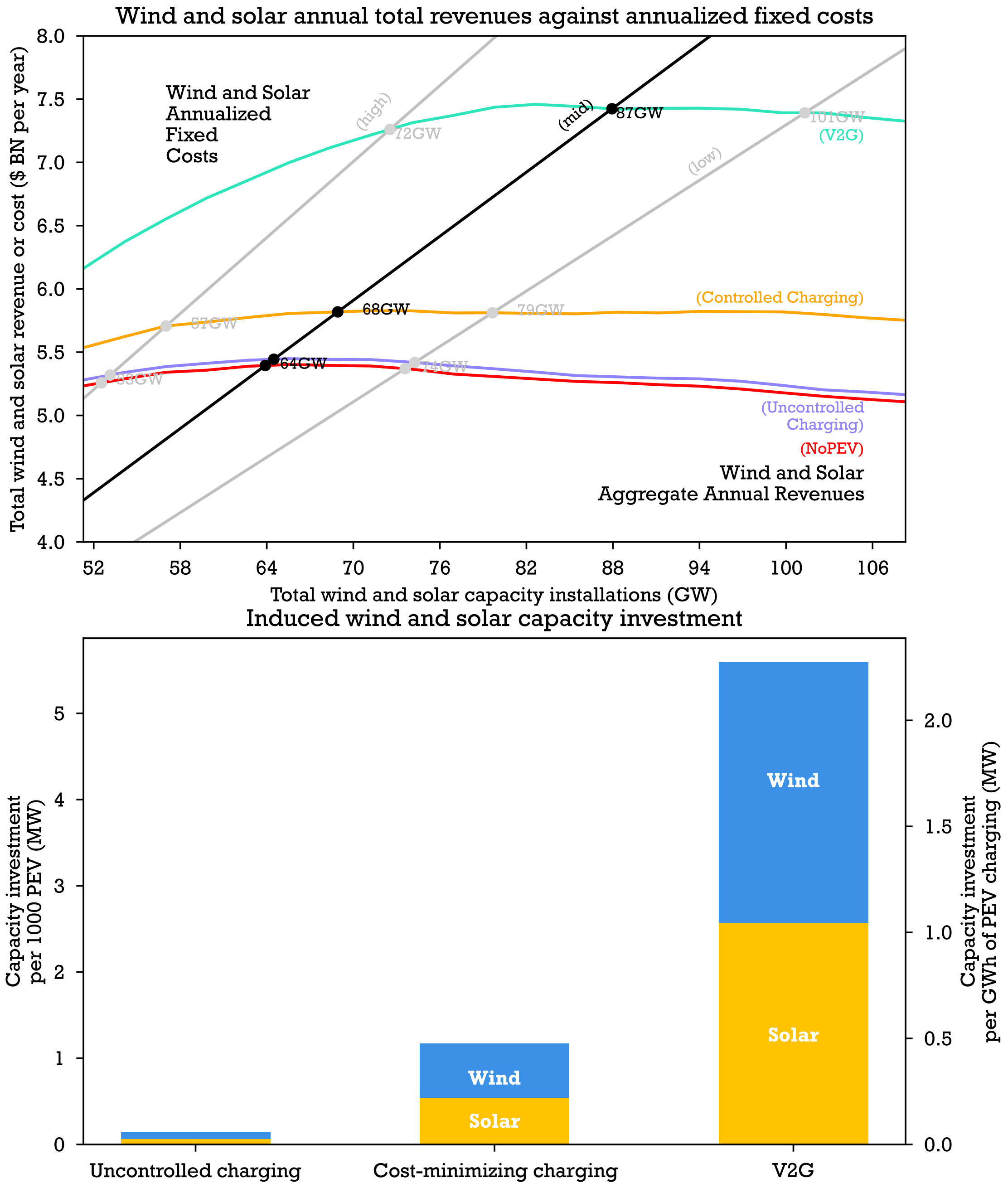

Figure S7: Effect of PEV charging load on the economics of wind and solar capacity investment. Top panel: Total revenues and total annualized fixed costs for solar and wind generators, by PEV and PEV charging intervention scenarios and by wind and solar fixed cost scenarios. **All results in this figure are run with 20% PEV penetration.** All other parameters are the same. Note: (high): conservative fixed cost scenario, (mid): base case fixed cost scenario, (low): optimistic fixed cost scenario. Bottom panel: Wind and solar capacity investment induced by PEV charging interventions, including uncontrolled charging (UC), cost-minimizing charging (CC) and vehicle-to-grid (V2G), relative to the NoPEV baseline scenario. The currency unit is 2024 USD. $ BN is short for billion USD. Further detailed methods are described in the method section.

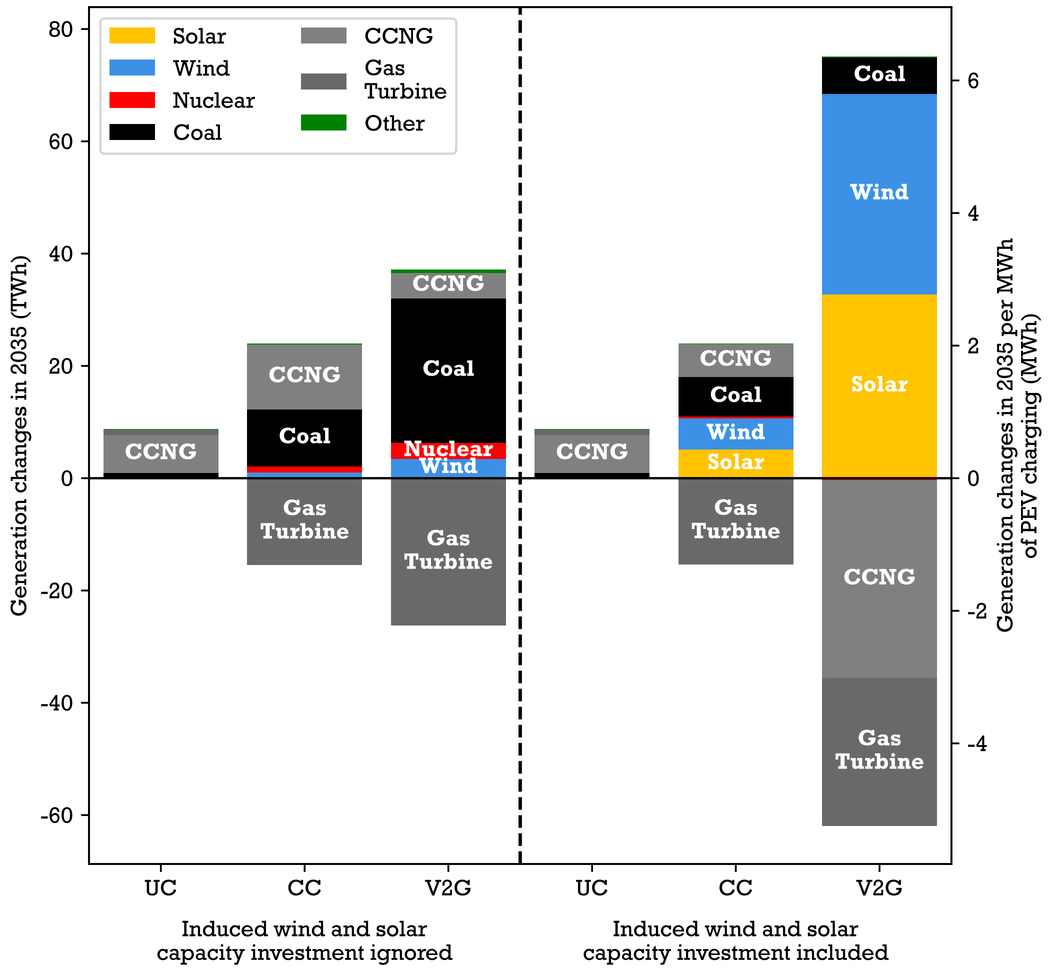

Figure S8: Effect of PEV charging load on power generation. Changes in annual generation by fuel type, relative to the NoPEV case, for each PEV charging scenario when ignoring versus including induced wind and solar capacity investment. **All results in this figure are run with 20% PEV penetration.** All other parameters are the same. When accounting for induced wind and solar investment, wind and solar capacity and therefore generation vary across PEV charging scenarios. 'CCNG' fuel type includes combined cycle natural gas generators. 'Other' fuel types include biomass, fossil waste, fuel cell, hydro, landfill gas, municipal solid waste, non-fossil waste, and oil or gas steam.

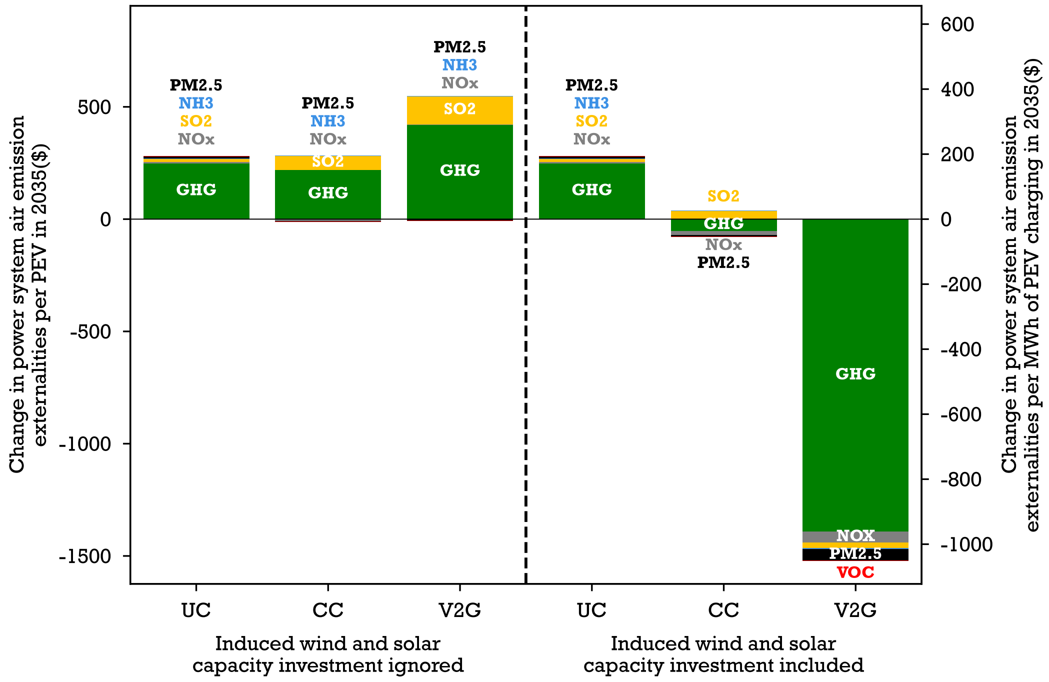

Figure S9: Effect of PEV charging on power system air emission externalities. Change in total power system air emission externalities per PEV and per MWh of PEV charging in 2035, relative to the NoPEV scenario, under each PEV charging scenario when ignoring versus including induced wind and solar capacity investment. The 'ignored' scenarios use generation portfolios given by PJM's grid planning study for 2035 [24]. The 'included' scenarios consider wind and solar capacity at maximum profitable capacity, as described in Method and Materials.

S3.4. Wind and solar capacity buildouts

To test the sensitivity of our results on the assumed fixed wind-to-solar capacity ratio, we run a high solar scenario, where solar capacity is increased by 10% and wind reduced by 10% compared with the base case, and a high wind scenario, where wind capacity is increased by 10% and solar reduced by 10% compared with the base case. The comparison with base case shows that while results are sensitive to wind and solar generation profiles, the conclusion still stands that PEVs under V2G can induce wind and solar capacity expansion, reduce overall power grid system costs and overall power grid emission externalities despite PEV charging.

S3.4.1. High solar scenario

Shown in Figure S10, with more solar and less wind, the total wind and solar capacity investment induced by V2G changes notably, but capacity investment induction benefits persists and are even higher than the base case. Under a high solar scenario, V2G increases the maximum profitable capacity of wind and solar capacity by 18 GW (a 26% increase) for a combined wind and solar capacity of 70 GW with no PEVs. The increases are 20% higher than under the base case: 15 GW (a 23% increase) increase for a combined capacity with no PEVs of 64 GW.

As a result of higher induced wind and solar capacity investment, the wind and solar generation changes induced by V2G under the high battery scenario are also higher than in the base case (shown in Figure S11). When induced capacity investment is considered, wind and solar generation increases (60 TWh) are 25% higher than under the base case (45 TWh).

When ignoring induced wind and solar capacity investment, total system costs under V2G are 5.5% lower than without PEVs (a $360 per PEV per year reduction). When accounting for induced wind and solar capacity investment, total system costs under V2G are 16% lower than without PEVs (a $1000 per PEV per year reduction). The cost reduction per PEV per year is 14% higher than in the base case (a $880 per PEV per year reduction).

Though results indicate the magnitude of emission externality reduction benefits is quite sensitive to wind and solar generation profiles, the benefits persist to be significant (shown in Figure S12). With higher solar capacity and lower wind capacity, total air emission externalities are reduced by $2300, 4.5% higher compared with the base case.

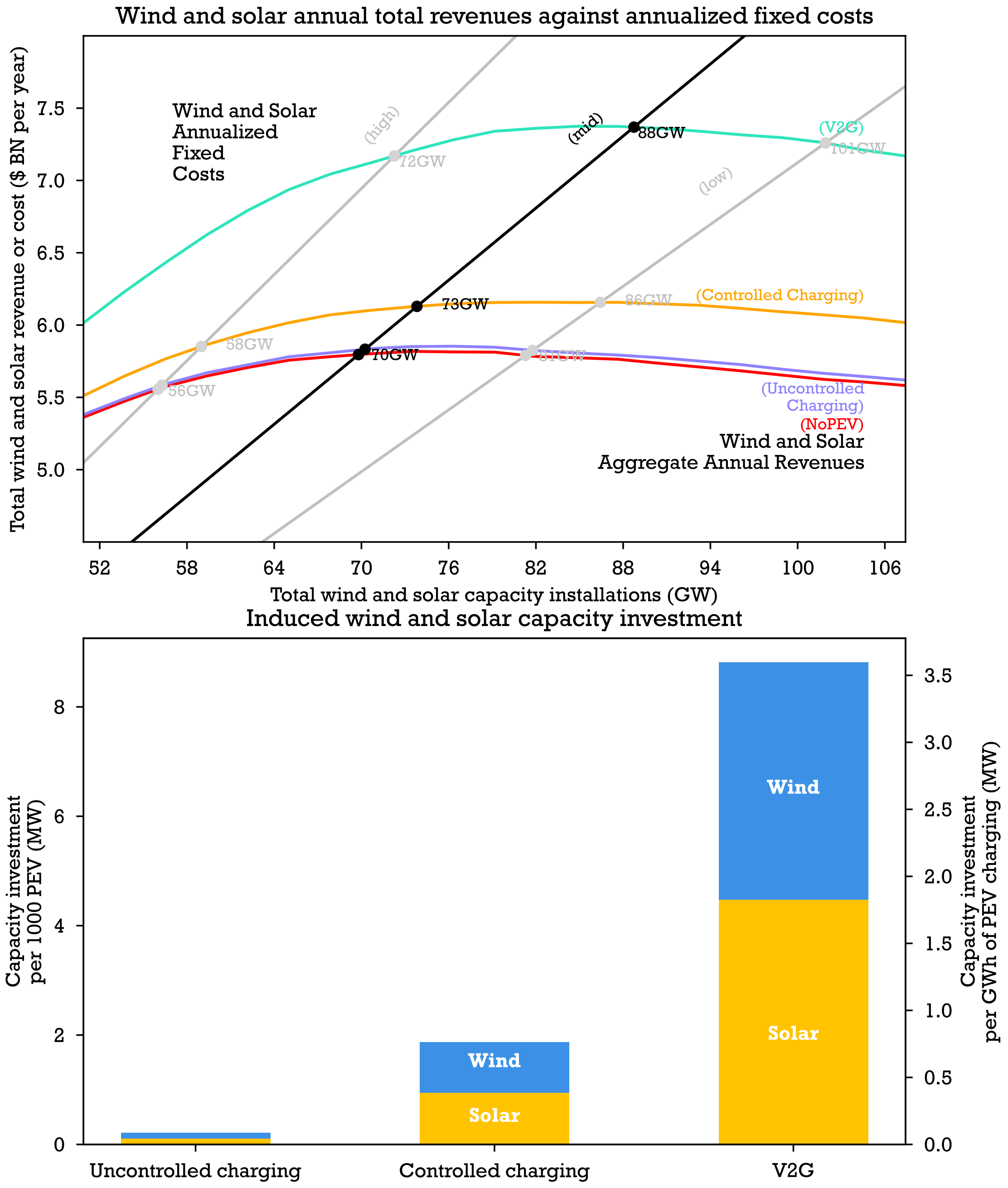

Figure S10: Effect of PEV charging load on the economics of wind and solar capacity investment. Top panel: Total revenues and total annualized fixed costs for solar and wind generators, by PEV and PEV charging intervention scenarios and by wind and solar fixed cost scenarios. **All results in this figure are run with additional solar capacity and lower wind capacity.** All other parameters are the same. Note: (high): conservative fixed cost scenario, (mid): base case fixed cost scenario, (low): optimistic fixed cost scenario. Bottom panel: Wind and solar capacity investment induced by PEV charging interventions, including uncontrolled charging (UC), cost-minimizing charging (CC) and vehicle-to-grid (V2G), relative to the NoPEV baseline scenario. The currency unit is 2024 USD. $ BN is short for billion USD. Further detailed methods are described in the method section.

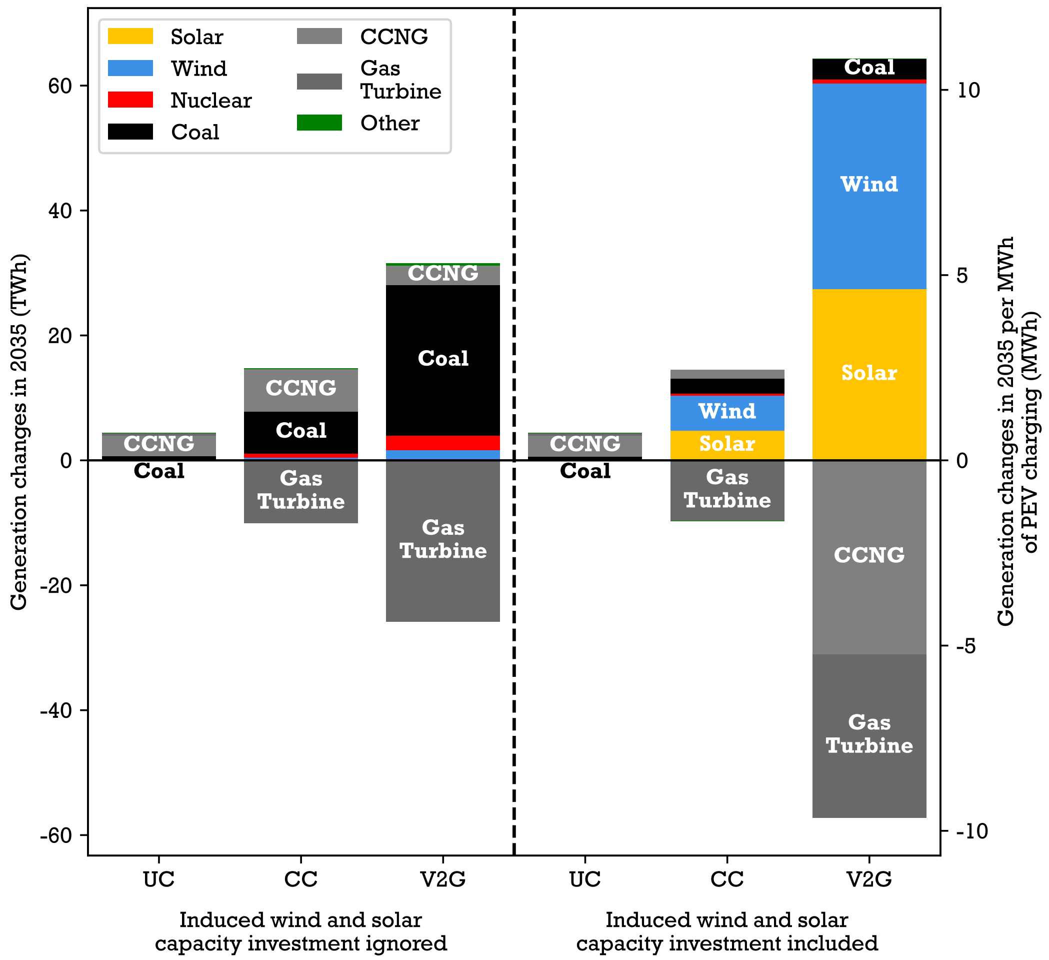

Figure S11: Effect of PEV charging load on power generation. Changes in annual generation by fuel type, relative to the NoPEV case, for each PEV charging scenario when ignoring versus including induced wind and solar capacity investment. **All results in this figure are run with additional solar capacity and lower wind capacity.** All other parameters are the same. When accounting for induced wind and solar investment, wind and solar capacity and therefore generation vary across PEV charging scenarios. 'CCNG' fuel type includes combined cycle natural gas generators. 'Other' fuel types include biomass, fossil waste, fuel cell, hydro, landfill gas, municipal solid waste, non-fossil waste, and oil or gas steam.

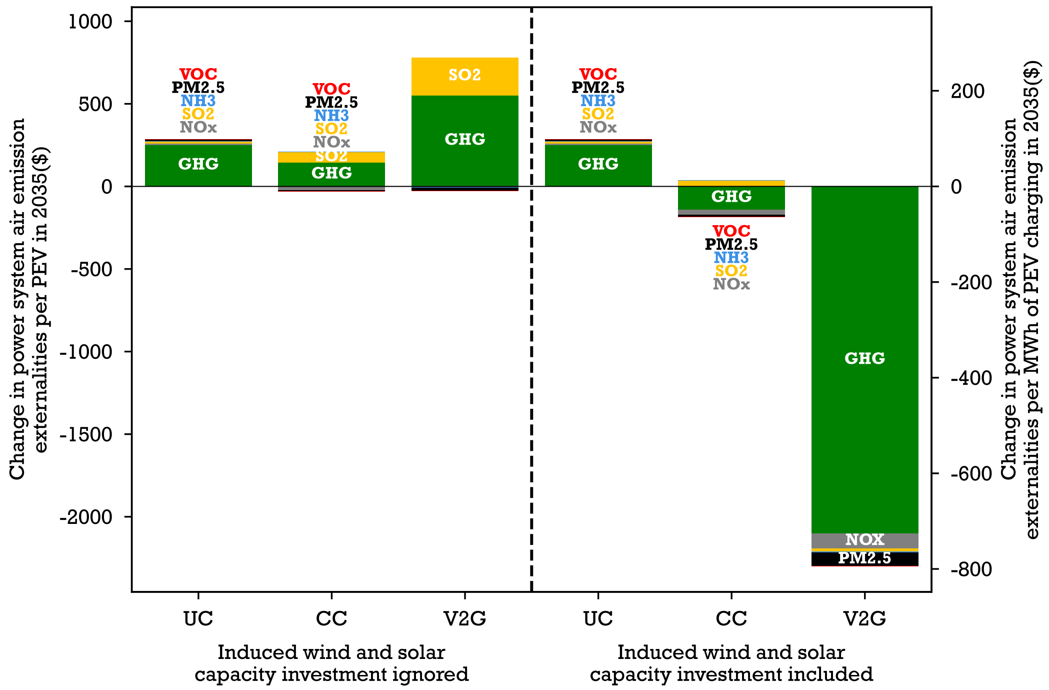

Figure S12: Effect of PEV charging on power system air emission externalities. Change in total power system air emission externalities per PEV and per MWh of PEV charging in 2035, relative to the NoPEV scenario, under each PEV charging scenario when ignoring versus including induced wind and solar capacity investment. The 'ignored' scenarios use generation portfolios given by PJM's grid planning study for 2035 [24]. The 'included' scenarios consider wind and solar capacity at maximum profitable capacity, as described in Method and Materials. **All results in this figure are run with additional solar capacity and lower wind capacity.**

S3.4.2. High wind scenario

Shown in Figure S13, with more wind and less solar, the total wind and solar capacity investment induced by V2G changes notably, but capacity investment induction benefits persist and are even higher than the base case. Under a high wind scenario, V2G increases the maximum profitable capacity of wind and solar capacity by 17 GW (a 24% increase) for a combined wind and solar capacity of 71 GW with no PEVs. The increases are 13% higher than under the base case: 15 GW (a 23% increase) increase for a combined capacity with no PEVs of 64 GW.

As a result of higher induced wind and solar capacity investment, the wind and solar generation changes induced by V2G under the high battery scenario are also higher than in the base case (shown in Figure S14). When induced capacity investment is considered, wind and solar generation increases (48 TWh) are 6.7% higher than under the base case (45 TWh).

When ignoring induced wind and solar capacity investment, total system costs under V2G are 5.5% lower than without PEVs (a $360 per PEV per year reduction). When accounting for induced wind and solar capacity investment, total system costs under V2G are 16% lower than without PEVs (a $1000 per PEV per year reduction). The cost reduction per PEV per year which is 14% higher than in the base case (a $880 per PEV per year reduction).

Shown in Figure S15. With higher solar capacity and lower wind capacity, total air emission externalities are reduced by $2300, 4% higher compared with the base case.

The two sets of sensitivity analysis results show that the magnitude of wind and solar capacity investment can be sensitive to wind and solar capacity buildout. As a result of different induced wind and solar capacity investments, system cost reduction benefits and emission reduction benefits can both be sensitive. However, in both sensitivity cases, all V2G benefits, including induced wind and solar capacity investment, system cost reduction benefits and emission reduction benefits, all persist and are higher than in the base case.

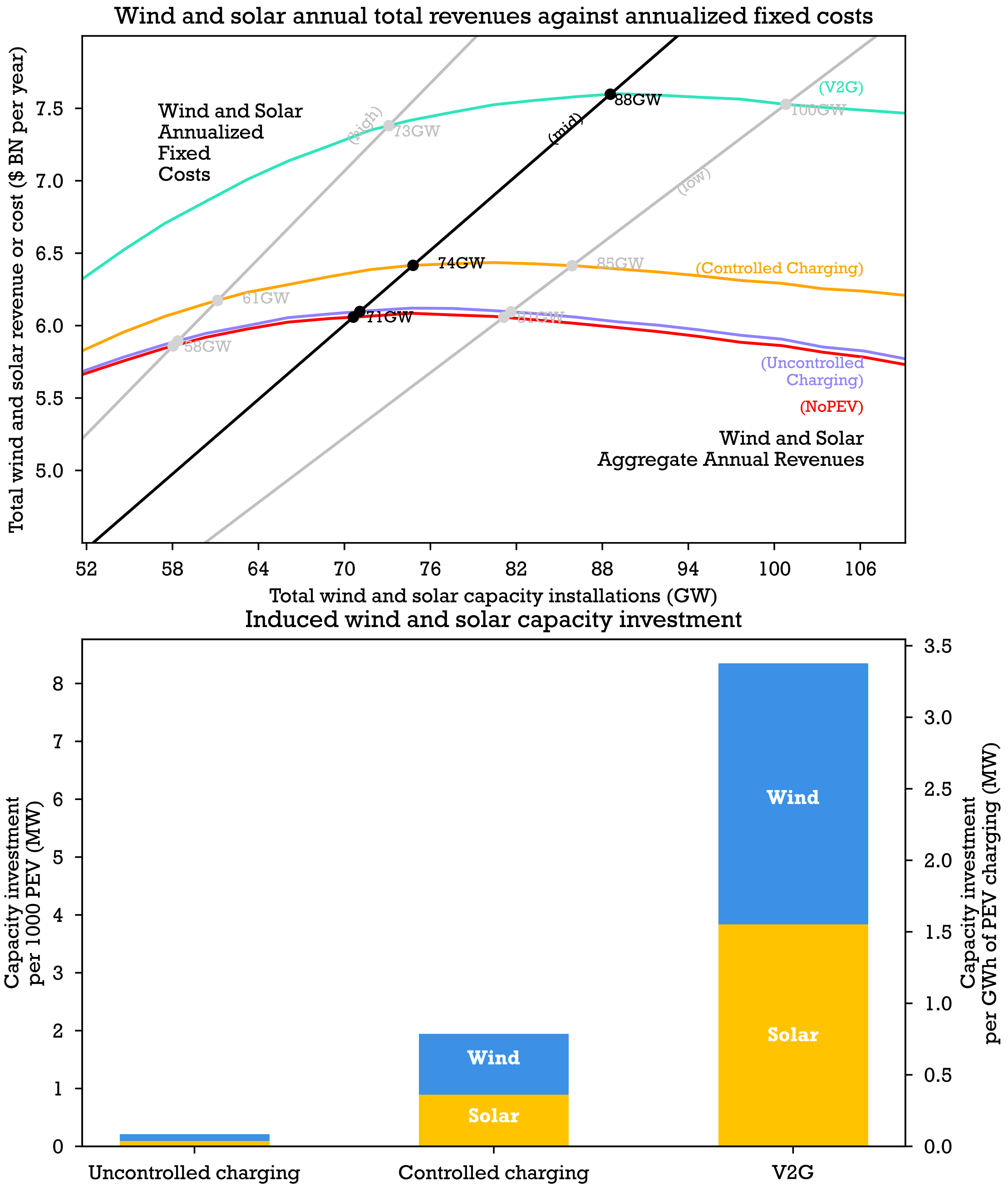

Figure S13: Effect of PEV charging load on the economics of wind and solar capacity investment. Top panel: Total revenues and total annualized fixed costs for solar and wind generators, by PEV and PEV charging intervention scenarios and by wind and solar fixed cost scenarios. **All results in this figure are run with additional wind capacity and lower solar capacity.** All other parameters are the same. Note: (high): conservative fixed cost scenario, (mid): base case fixed cost scenario, (low): optimistic fixed cost scenario. Bottom panel: Wind and solar capacity investment induced by PEV charging interventions, including uncontrolled charging (UC), cost-minimizing charging (CC) and vehicle-to-grid (V2G), relative to the NoPEV baseline scenario. The currency unit is 2024 USD. $ BN is short for billion USD. Further detailed methods are described in the method section.

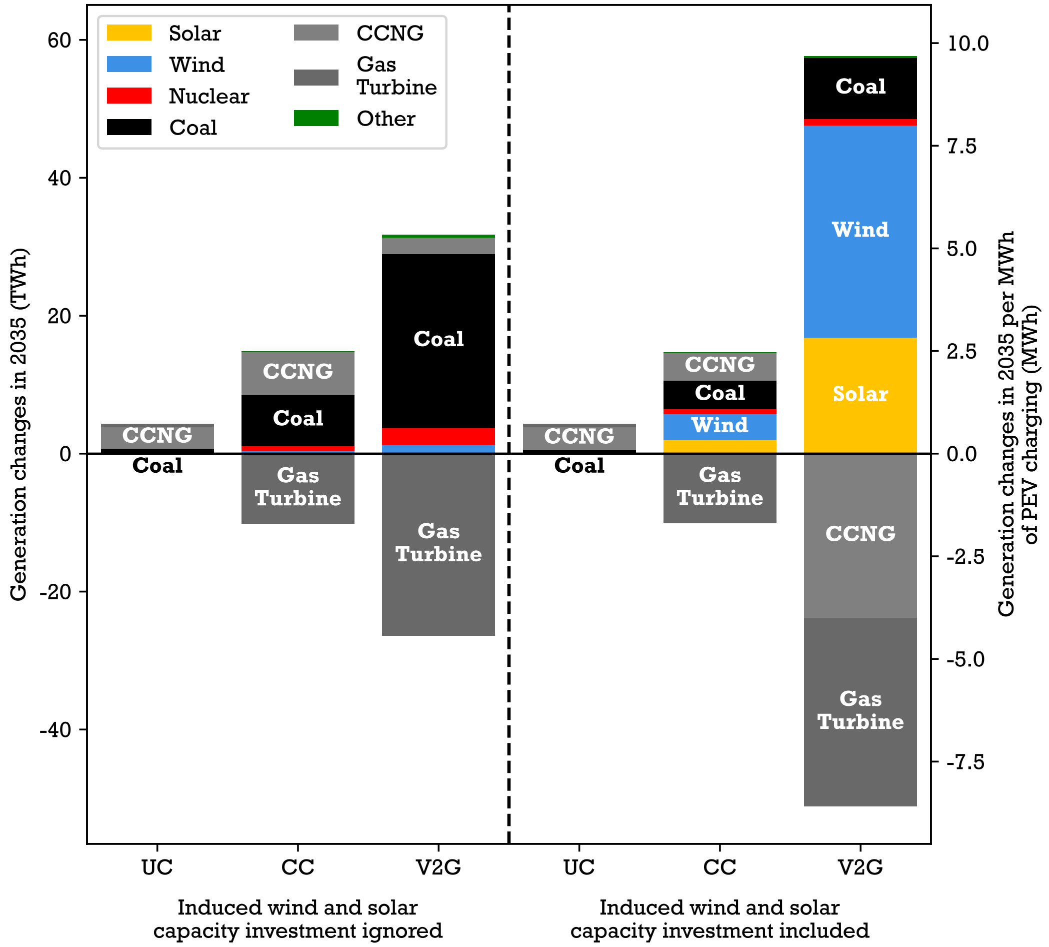

Figure S14: Effect of PEV charging load on power generation. Changes in annual generation by fuel type, relative to the NoPEV case, for each PEV charging scenario when ignoring versus including induced wind and solar capacity investment. **All results in this figure are run with additional wind capacity and lower solar capacity.** All other parameters are the same. When accounting for induced wind and solar investment, wind and solar capacity and therefore generation vary across PEV charging scenarios. 'CCNG' fuel type includes combined cycle natural gas generators. 'Other' fuel types include biomass, fossil waste, fuel cell, hydro, landfill gas, municipal solid waste, non-fossil waste, and oil or gas steam.

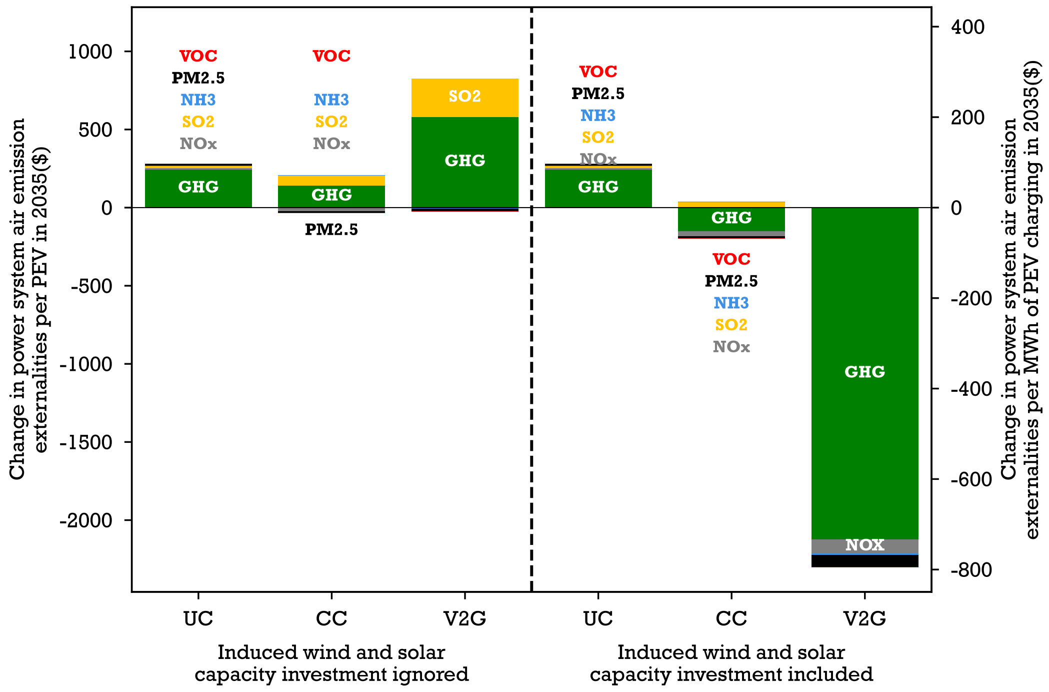

Figure S15: Effect of PEV charging on power system air emission externalities. Change in total power system air emission externalities per PEV and per MWh of PEV charging in 2035, relative to the NoPEV scenario, under each PEV charging scenario when ignoring versus including induced wind and solar capacity investment. The 'ignored' scenarios use generation portfolios given by PJM's grid planning study for 2035 [24]. The 'included' scenarios consider wind and solar capacity at maximum profitable capacity, as described in Method and Materials. **All results in this figure are run with additional solar capacity and lower wind capacity.**

S4. Methods

S4.1. Variable renewable energy cost estimation

We use cost modeling results from National Renewable Energy Laboratory's Annual Technology Baseline (ATB) database to assess fixed costs of solar and wind generators [25]. 2035 Conservative, Moderate, and Advanced scenarios in ATB correspond to Conservative, Base case, and Optimistic scenarios in our study. Annualized capital costs are calculated with an interest rate of 5% and a capital recovery term of 30 years. The main results reported in the manuscript are based on the Base case scenario. Induced wind and solar capacity is also reported for Conservative and Advanced scenarios. The costs of three scenarios are shown in Table S2.

Table S2: Capital expenditures and fixed O&M costs of wind and power sources in 2024 USD, under different cost scenarios [25]

Technology		Optimistic	Base case	Conservative
On-shore wind	Capital expenditures (USD/kW)	1034	1093	1231
	Annual O&M (USD/kW)	21.6	26.1	28.1
Off-shore wind	Capital expenditures (USD/kW)	2397	2709	3414
	Annual O&M (USD/kW)	75.1	84.5	104.8
Solar	Capital expenditures (USD/kW)	632	829	1101
	Annual O&M (USD/kW)	13.1	15.5	18.9

S4.2. PJM System Topography

The original modeling of the PJM Interconnection as 5 transmission constrained regions (TCR) was developed in Lueken et al. 2014 and Weis et al. 2014 [12], [26], which was later updated by Bruchon et al. 2024 [27]. The TCRs and transmission interfaces connecting TCRs are shown in Figure S16. Transmission lines are aggregated into 5 inter-regional transmission interfaces, shown in Figure S17. Transmission capacity of each interfaces are extracted from PJM website (PJM Data Miner).

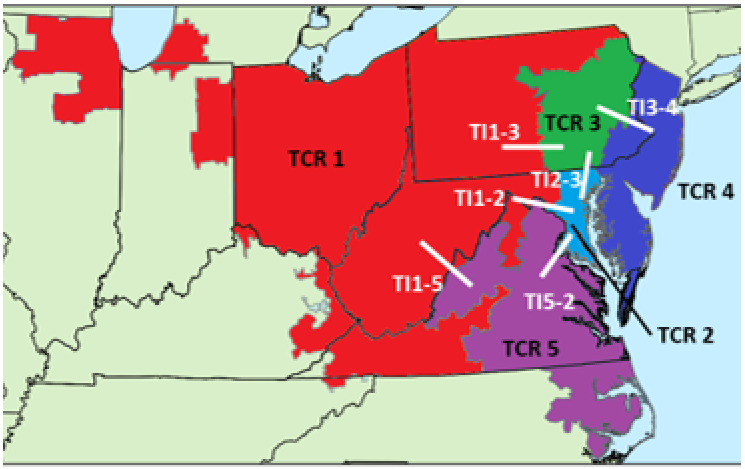

Figure S16: Map of Transmission Constrained Regions (TCRs) and Transmission Interconnections (TIs).

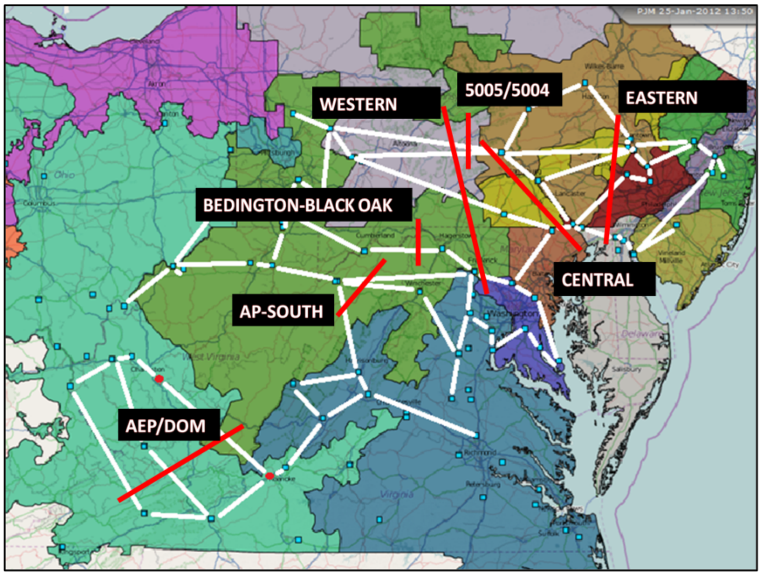

Figure S17: Map of PJM 500kV transmission lines (white lines) and transmission interfaces (red lines). Most interfaces contain multiple 500kV lines. The map illustrates model representation of PJM in Bruchon et al. 2024.

S4.3. Power system model formulation

We in this study updated and adapted the unit commitment economic dispatch model formulation of Weis. et al. 2016 and Bruchon et al. 2024 to enable V2G functionalities [27], [28]. The mixed integer optimization problem is formulated as follows in Table S5 and Table S4:

This study models two reserve products: regulation reserve (5min) and spinning reserve (10min). For the calculation of these requirements, we adopt the parameters developed by the National Renewable Energy Laboratory (NREL) ReEDS model [29]. The reserve provision costs account for variable costs incurred by degraded heat rate and other factors, and we adopt the parameters used by NREL's ReEDS model and Craig et al. 2017 (seen in Table tb:rescost) [29], [30].

Table S3: Nomenclature: sets, decision variables, and input parameters.The nomenclature is for Table S4. The problem formulation describes the unit commitment economic dispatch model that simulates hourly simulation of PJM's power system for one year.

Label	Type	Description
$\mathcal{I}$	Set	Generating units
$\mathcal{K}$	Set	Storage units
$\mathcal{R}$	Set	Transmission constraint regions (TCRs)
$\mathcal{T}$	Set	Timesteps
$\mathcal{V}$	Set	Plug-in electric vehicle schedule groups
$c_{v,r,t}^{\text{VEH}}$	Variable	Charge level (MWh) of vehicle schedule group v in TCR r at time t
$c_{k,t}^{\text{STR}}$	Variable	Charge level (MWh) of storage unit k at time t
$p_{i,t}^{\text{GEN}}$	Variable	Power generated by unit i at time t
$p_{r,t}^{\text{SGEN}}$	Variable	Power generated by solar PV generators in TCR r at time t
$p_{r,t}^{\text{WGEN}}$	Variable	Power generated by wind generators in TCR r at time t
$p_{k,t}^{\text{STRD}}$	Variable	Power discharged (+) by storage unit k at time t
$p_{k,t}^{\text{STRC}}$	Variable	Power charged (+) by storage unit k at time t
$p_{r,r',t}^{\text{RR}}$	Variable	Power imported (+) from TCR r' to TCR $r (r \neq r')$ at time t
$p_{v,r,t}^{\text{VR}}$	Variable	Power charged (+) by vehicle schedule group v in TCR r at time t
$p_{v,r,t}^{\text{DR}}$	Variable	Power discharged (+) by vehicle schedule group v in TCR r at time t
$a_{i,t}^{\text{GEN}}$	Variable	Reserve provided by generator unit i at time t
$a_{k,t}^{\text{STR}}$	Variable	Reserve provided by storage unit k at time t
$a_{v,r,t}^{\text{VR}}$	Variable	Reserve provided by vehicle schedule group v in TCR r at time t
$a_{i,t}^{\text{GENSP}}$	Variable	Spinning reserve provided by generator unit i at time t
$a_{k,t}^{\text{STRSP}}$	Variable	Spinning reserve provided by storage unit k at time t
$a_{v,r,t}^{\text{VRSP}}$	Variable	Spinning reserve provided by vehicle schedule group v in TCR r at time t
$s_{i,t}^{\text{STARTUP}}$	Variable	Slack variable for startup cost
$u_{i,t}$	Variable	Binary variable that is equal to 1 if unit i is online in period t and 0 otherwise
$A_{v,t}$	Parameter	Availability of vehicle schedule group v to charge at time t (%)
B_v	Parameter	Battery capacity (MWh) of vehicle grouping v
c_k^{STRMAX}	Parameter	Max charge level (MWh) of storage unit k
c_k^{STRMIN}	Parameter	Min charge level (MWh) of storage unit k
C^{STR}	Parameter	Reserve provision cost of storage unit
C^{VEH}	Parameter	Reserve provision cost of PEV
C_i^{VARGEN}	Parameter	Variable cost of generating unit i
C_i^{STARTGEN}	Parameter	Startup cost of generating unit i
$p_{r,t}^{\text{SGENMAX}}$	Parameter	Maximum power generated by solar PV generators in TCR r at time t
$p_{r,t}^{\text{WGENMAX}}$	Parameter	Maximum power generated by wind generators in TCR r at time t
$P_{r,t}$	Parameter	Power demand in TCR r at time t
$p_{r,r',t}^{\text{RRMAX}}$	Parameter	Max power flow on interface (r, r') at time t
$M_{v,t}$	Parameter	Miles traveled per car in vehicle schedule group v at time t
$N_{v,r}$	Parameter	Number of vehicles in schedule group v in TCR r
p_i^{GENMIN}	Parameter	Min generation from unit i at time t
p_i^{GENMAX}	Parameter	Max generation from unit i at time t
p_k^{STRMAX}	Parameter	Max discharge rate from storage unit k
R_i	Parameter	Ramp rate limit of unit i
R_v^{MAXCHG}	Parameter	Max charge rate (MW) of vehicle schedule group v
T_i^{UPMIN}	Parameter	Minimum uptime of unit i
T_i^{DTMIN}	Parameter	Minimum downtime of unit i
C_i^{GENRES}	Parameter	Reserve provision cost of unit i
T_i^{UPSTART}	Parameter	Number of timesteps unit i has been online at initial timestep $t = 1$
T_i^{DTSTART}	Parameter	Number of timesteps unit i has been offline at initial timestep $t = 1$
T_v^{DEPART}	Parameter	Timestep at which vehicle schedule group v departs home
η_v^{CHG}	Parameter	Charging and discharging efficiency (%) of vehicle schedule group v
η_k^{STR}	Parameter	Efficiency (%) of storage unit k
η_v^{VEH}	Parameter	Driving efficiency (MWh/mile) of vehicle schedule group v
η^{TR}	Parameter	Transmission efficiency (%)
p_r^{S}	Parameter	Solar PV capacity of region r (MW)
ϕ^{REG}	Parameter	Ratio of load regulation reserve requirement to load (%)
ϕ^{WIND}	Parameter	Ratio of wind regulation reserve requirement to regional wind generation (%)
ϕ^{SOL}	Parameter	Ratio of solar PV regulation reserve requirement to regional solar PV installed capacity (%)
ϕ^{SPIN}	Parameter	Ratio of spinning reserve requirement to load (%)

Table S4: Optimization problem formulation. The problem formulation describes the unit commitment economic dispatch model that simulates hourly simulation of PJM's power system for one year.

$$\text{Minimize } Z = \sum_{t \in \mathcal{T}} \sum_{i \in I_r} \left(C_i^{\text{VARGEN}} p_{i,t}^{\text{GEN}} + s_{i,t}^{\text{STARTUP}} + C_i^{\text{GENRES}} a_{i,t}^{\text{GEN}} \right) + \sum_{t \in \mathcal{T}} \sum_{k \in \mathcal{K}} C^{\text{STR}} a_{k,t}^{\text{STR}} + \sum_{t \in \mathcal{T}} \sum_{v \in \mathcal{V}} \sum_{r \in \mathcal{R}} C^{\text{VEH}} a_{v,r,t}^{\text{VEH}}$$

Subject to

Generator constraints:

Constraint	Domain	Description	No.
$a_{i,t}^{\text{GEN}} \leq 1/12 * \left(p_{i,t-1} + R_i u_{i,t} + p_i^{\text{GENMIN}}(u_{i,t} - u_{i,t-1}) - p_{i,t} \right) - a_{i,t}^{\text{GENSP}}$	$\forall i \in \mathcal{I} \neq 1, \forall t \in \mathcal{T}$	Generator regulation reserve offer constraints	(1)
$a_{i,t}^{\text{GEN}} \leq 1/12 * \left(p_i^{\text{GENMAX}} - p_{i,t} \right) - a_{i,t}^{\text{GENSP}}$	$\forall i \in \mathcal{I} \neq 1, \forall t \in \mathcal{T}$	Generator regulation reserve offer constraints	(2)
$a_{i,t}^{\text{GEN}} \leq 1/12 * \left(p_i^{\text{GENMAX}} u_{i,t} \right) - a_{i,t}^{\text{GENSP}}$	$\forall i \in \mathcal{I} \neq 1, \forall t \in \mathcal{T}$	Generator regulation reserve offer constraints	(3)
$a_{i,t}^{\text{GENSP}} \leq 1/6 * \left(p_{i,t-1} + R_i u_{i,t} + p_i^{\text{GENMIN}}(u_{i,t} - u_{i,t-1}) - p_{i,t} \right) - a_{i,t}^{\text{GENSP}}$	$\forall i \in \mathcal{I} \neq 1, \forall t \in \mathcal{T}$	Generator spinning reserve offer constraints	(4)
$a_{i,t}^{\text{GENSP}} \leq 1/6 * \left(p_i^{\text{GENMAX}} - p_{i,t} \right) - a_{i,t}^{\text{GEN}}$	$\forall i \in \mathcal{I} \neq 1, \forall t \in \mathcal{T}$	Generator spinning reserve offer constraints	(5)
$a_{i,t}^{\text{GENSP}} \leq 1/6 * \left(p_i^{\text{GENMAX}} u_{i,t} \right) - a_{i,t}^{\text{GEN}}$	$\forall i \in \mathcal{I} \neq 1, \forall t \in \mathcal{T}$	Generator spinning reserve offer constraints	(6)
$s_{i,t}^{\text{STARTUP}} \geq \left(u_{i,t} - u_{i,t-1} \right) C_i^{\text{STARTGEN}}$	$\forall i \in \mathcal{I}, \forall t \in \mathcal{T}$	Slack variable reflects startup decision	(7)
$s_{i,t}^{\text{STARTUP}} \geq 0$	$\forall i \in \mathcal{I}, \forall t \in \mathcal{T}$	Slack variable is nonnegative	(8)
$p_i^{\text{GENMIN}} u_{i,t} \leq p_{i,t} \leq p_i^{\text{GENMAX}} u_{i,t}$	$\forall i \in \mathcal{I}, \forall t \in \mathcal{T}$	Min and max output of online generators	(9)
$p_{i,t}^{\text{GEN}} \leq p_{i,t-1}^{\text{GEN}} + R_i u_{i,t-1} + p_i^{\text{GENMIN}} \left(u_{i,t} - u_{i,t-1} \right)$	$\forall i \in \mathcal{I}, \forall t \in \mathcal{T}$	Ramp rate limit	(10)
$p_{i,t-1}^{\text{GEN}} \leq p_{i,t}^{\text{GEN}} + R_i u_{i,t} + p_i^{\text{GENMIN}} \left(u_{i,t-1} - u_{i,t} \right)$	$\forall i \in \mathcal{I}, \forall t \in \mathcal{T}$	Ramp rate limit	(11)
$\sum_{t=2}^{T_i^{\text{UPMIN}} - T_i^{\text{UPSTART}}} \left(1 - u_{i,t} \right) = 0$	$\forall i \in \mathcal{I}$	Minimum uptime (first timesteps)	(12)
$\sum_{t'=t}^{t + T_i^{\text{UPMIN}} - 1} u_{i,t'} \geq T_i^{\text{UPMIN}} \left(u_{i,t} - u_{i,t-1} \right)$	$\forall i \in \mathcal{I}, \forall t : T_i^{\text{UPMIN}} - T_i^{\text{UPSTART}} + 1 \leq t \leq \lvert \mathcal{T} \rvert - T_i^{\text{UPMIN}} + 1$	Minimum uptime (middle timesteps)	(13)
$\sum_{t=t}^{T} u_{i,t'} \geq (T - t) \left(u_{i,t} - u_{i,t-1} \right)$	$\forall i \in \mathcal{I}, \forall t \in \lvert \mathcal{T} \rvert - T_i^{\text{UPMIN}} + 2 \ldots$	Minimum uptime (final timesteps)	(14)
$\sum_{t=2}^{T_i^{\text{DTMIN}} - T_i^{\text{DTSTART}}} u_{i,t} = 0$	$\forall i \in \mathcal{I}$	Minimum downtime (first timesteps)	(15)
$\sum_{t'=t}^{t + T_i^{\text{DTMIN}} - 1} 1 - u_{i,t'} \geq T_i^{\text{DTMIN}} \left(u_{i,t-1} - u_{i,t} \right)$	$\forall i \in \mathcal{I}, \forall t : T_i^{\text{DTSTART}} + 1 \leq t \leq \lvert \mathcal{T} \rvert - T_i^{\text{DTSTART}} + 1$	Minimum downtime (middle timesteps)	(16)
$\sum_{t'=t}^{T} 1 - u_{i,t'} \geq (\lvert \mathcal{T} \rvert - t) \left(u_{i,t-1} - u_{i,t} \right)$	$\forall i \in \mathcal{I}, \forall t : \lvert \mathcal{T} \rvert - T_i^{\text{DTSTART}} + 2 \leq t \leq \lvert \mathcal{T} \rvert$	Minimum downtime (final timesteps)	(17)

Table S5: Optimization problem formulation (continued).

$$\text{Minimize } Z = \sum_{t\in\mathcal{T}}\sum_{i\in I_r}\left(C_i^{\text{VARGEN}} p_{i,t}^{\text{GEN}} + s_{i,t}^{\text{STARTUP}} + C_i^{\text{GENRES}} a_{i,t}^{\text{GEN}}\right) + \sum_{t\in\mathcal{T}}\sum_{k\in\mathcal{K}} C_k^{\text{STR}} a_{k,t}^{\text{STR}} + \sum_{t\in\mathcal{T}}\sum_{v\in\mathcal{V}}\sum_{r\in\mathcal{R}} C^{\text{VEH}} a_{v,r,t}^{\text{VEH}}$$

Subject to

System constraints:

$$P_{r,t} = \sum_{i\in\mathcal{I}_r} p_{i,t}^{\text{GEN}} + \sum_{k\in\mathcal{K}_r} p_{i,t}^{\text{STRD}} - \sum_{k\in\mathcal{K}_r} p_{i,t}^{\text{STRC}} - \sum_{r'\in\mathcal{R}\neq r}(p_{r',r,t}^{\text{RR}}) + \sum_{r'\in\mathcal{R}\neq r}(p_{r,r',t}^{\text{RR}}\eta^{\text{TR}}) + p_{r,t}^{\text{SGEN}} + p_{r,t}^{\text{WGEN}} - \sum_{v\in\mathcal{V}_r} p_{v,r,t}^{\text{VR}} + \sum_{v\in\mathcal{V}_r} p_{v,r,t}^{\text{DR}} \qquad \forall r\in\mathcal{R}, t\in\mathcal{T} \tag{18}$$

Demand must equal supply

$$-p_{r,r',t}^{\text{RRMAX}} \le p_{r,r',t}^{\text{RR}} \le p_{r,r',t}^{\text{RRMAX}} \qquad \forall r\in\mathcal{R}, r'\in\mathcal{R}\neq r, t\in\mathcal{T} \tag{19}$$

Inter-region power flow limits

$$0 \le p_{r,t}^{\text{SGEN}} \le p_{r,t}^{\text{SGENMAX}} \qquad \forall r\in\mathcal{R}, t\in\mathcal{T} \tag{20}$$

Regional solar PV output limits

$$0 \le p_{r,t}^{\text{WGEN}} \le p_{r,t}^{\text{WGENMAX}} \qquad \forall r\in\mathcal{R}, t\in\mathcal{T} \tag{21}$$

Regional wind generator output limits

$$\phi^{SPIN} * P_{r,t} = \sum_{i\in\mathcal{I}_r} a_{i,t}^{\text{GENSP}} + \sum_{k\in\mathcal{K}_r} a_{k,t}^{\text{STRSP}} + \sum_{v\in\mathcal{V}_r} a_{v,r,t}^{\text{VRSP}} \qquad \forall r\in\mathcal{R}, t\in\mathcal{T} \tag{22}$$

Regional spinning reserve requirement must be met

$$\phi^{REG} * P_{r,t} + \phi^{\text{WIND}} * p_{r,t}^{\text{WGEN}} + \phi^{\text{SOL}} * p^{\text{S}} = \sum_{i\in\mathcal{I}_r} a_{i,t}^{\text{GEN}} + \sum_{k\in\mathcal{K}_r} a_{k,t}^{\text{STR}} + \sum_{v\in\mathcal{V}_r} a_{v,r,t}^{\text{VR}} \qquad \forall r\in\mathcal{R}, t\in\mathcal{T} \tag{23}$$

Regional regulation reserve requirement must be met

Storage constraints:

$$c_{k,t+1}^{\text{STR}} = c_{k,t}^{\text{STR}} + p_{k,t}^{\text{STRC}}\eta^{STR} - p_{k,t}^{\text{STRD}}/\eta^{STR} \qquad \forall k\in\mathcal{K}, \forall t\in\mathcal{T} \tag{24}$$

Storage state of charge

$$c_k^{\text{STRMIN}} \le c_{k,t}^{\text{STR}} \le c_k^{\text{STRMAX}} \qquad \forall k\in\mathcal{K}, \forall t\in\mathcal{T} \tag{25}$$

Storage unit capacity

$$0 \le p_{k,t}^{\text{STRD}}, p_{k,t}^{\text{STRC}} \le p_k^{\text{STRMAX}} \qquad \forall k\in\mathcal{K}, \forall t\in\mathcal{T} \tag{26}$$

Max charge and discharge of storage units

$$a_{k,t}^{\text{STRSP}} \le p_k^{\text{STRMAX}} - p_{k,t}^{\text{STRD}} + p_{k,t}^{\text{STRC}} - a_{k,t}^{\text{STR}} \qquad \forall k\in\mathcal{K}, \forall t\in\mathcal{T} \tag{27}$$

Max regulation and spinning reserve offer of storage units

$$a_{k,t}^{\text{STRSP}} \le c_{k,t}^{\text{STR}} - a_{k,t}^{\text{STR}} \qquad \forall k\in\mathcal{K}, \forall t\in\mathcal{T} \tag{28}$$

Max regulation and spinning reserve offer of storage units

PEV constraints:

$$c_{v,r,t+1}^{\text{VEH}} = c_{v,r,t}^{\text{CHG}} - p_{v,r,t}^{\text{VR}}\eta_v^{\text{VEH}} - N_{v,r}M_{v,t}\eta_v^{\text{VEH}} \qquad \forall v\in\mathcal{V}, \forall r\in\mathcal{R}, \forall t\in\mathcal{T} \tag{29}$$

PEV state of charge

$$10\% N_{v,r}B_v \le c_{v,r,t}^{\text{VEH}} \le 90\% N_{v,r}B_v \qquad \forall v\in\mathcal{V}, \forall r\in\mathcal{R}, \forall t\in\mathcal{T} \tag{30}$$

PEV battery capacity

$$c_{v,r,t}^{\text{VEH}} = 90\% N_{v,r}B_v \qquad \forall v\in\mathcal{V}, \forall r\in\mathcal{R}, \forall t = T_v^{\text{DEPART}} \tag{31}$$

PEVs fully charged when departing home

$$c_{v,r,t+1}^{\text{VEH}} = c_{v,r,t}^{\text{CHG}} - p_{v,r,t}^{\text{VR}}/\eta_v^{\text{CHG}} + p_{v,r,t}^{\text{DR}} * \eta_v^{\text{CHG}} N_{v,r}M_{v,t}\eta_v^{\text{VEH}} \qquad \forall v\in\mathcal{V}, \forall r\in\mathcal{R}, \forall t\in\mathcal{T} \tag{32}$$

PEV state of charge

$$0 \le p_{v,r,t}^{\text{VR}}, p_{v,r,t}^{\text{DR}} \le N_{v,r}A_{v,t}R_v^{\text{MAXCHG}} \qquad \forall v\in\mathcal{V}, \forall r\in\mathcal{R}, \forall t\in\mathcal{T} \tag{33}$$

PEV charge rate limit

$$0 \le c_{v,r,t}^{\text{VEH}} \le N_{v,r}B_v \qquad \forall v\in\mathcal{V}, \forall r\in\mathcal{R}, \forall t\in\mathcal{T} \tag{34}$$

PEV battery capacity

$$a_{v,r,t}^{\text{VRSP}} \le N_{v,r}A_{v,t}R_v^{\text{MAXCHG}} - p_{v,r,t}^{\text{DR}} + p_{v,r,t}^{\text{VR}} - a_{v,r,t}^{\text{VR}} \qquad \forall r\in\mathcal{R}, \forall v\in\mathcal{V}$$

Max regulation and spinning reserve offer of vehicle fleets

$$a_{v,r,t}^{\text{VRSP}} \le c_{v,r,t}^{\text{VEH}} - a_{v,r,t}^{\text{VR}} \qquad \forall r\in\mathcal{R}, \forall v\in\mathcal{V}, \forall t\in\mathcal{T} \tag{35}$$

(36)

Table S6: Reserve provisional costs of different reserve providers by fuel type or technology type

Fuel and technology type	Coal	Combined Cycle Natural Gas	Gas/Oil steam	Storage	Storage by PEV
Cost($/MWh)	10	6	4	2	2

182 S5. More results

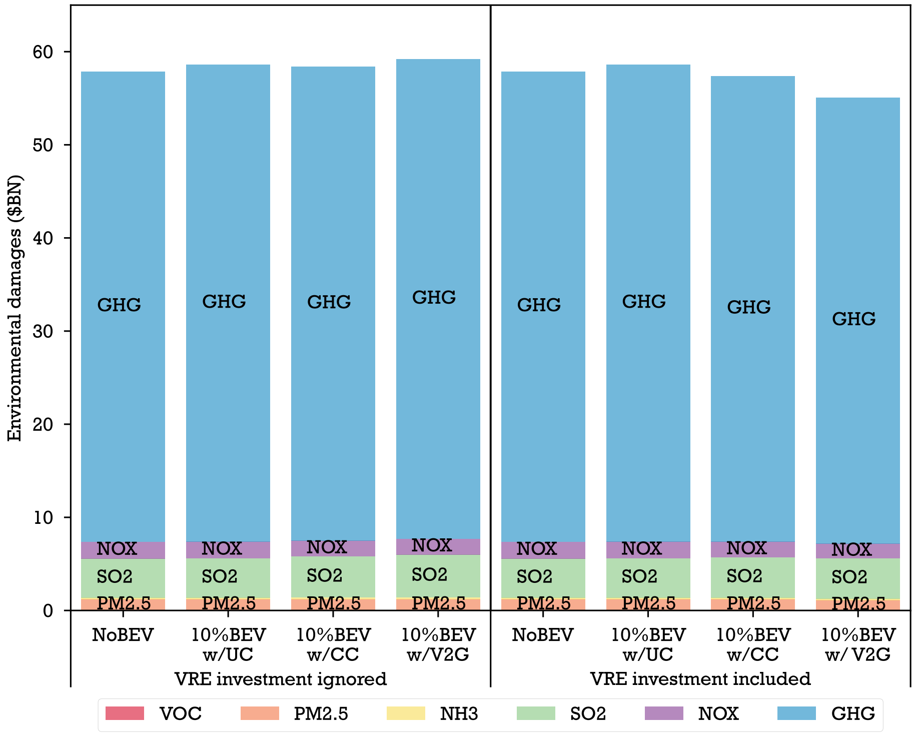

Figure S18: Annual environmental externalities of GHG emissions and pollution, by different PEV and PEV charging intervention scenarios and analysis approaches. Wind and power capacity varies across PEV and PEV charging intervention scenarios in Expansion scenarios, as PEV and PEV charging intervention affects the maximum profitable wind and power capacity installation. Environmental externalities for GHG emissions are calculated with social cost of carbon of $204/ton CO_2e in 2023, published by US Environment Protection Agency[31]. The results with a lower cost of carbon of $51/ton CO_2e can be found in SI. The AP3 model is used to calculate those of pollutants.

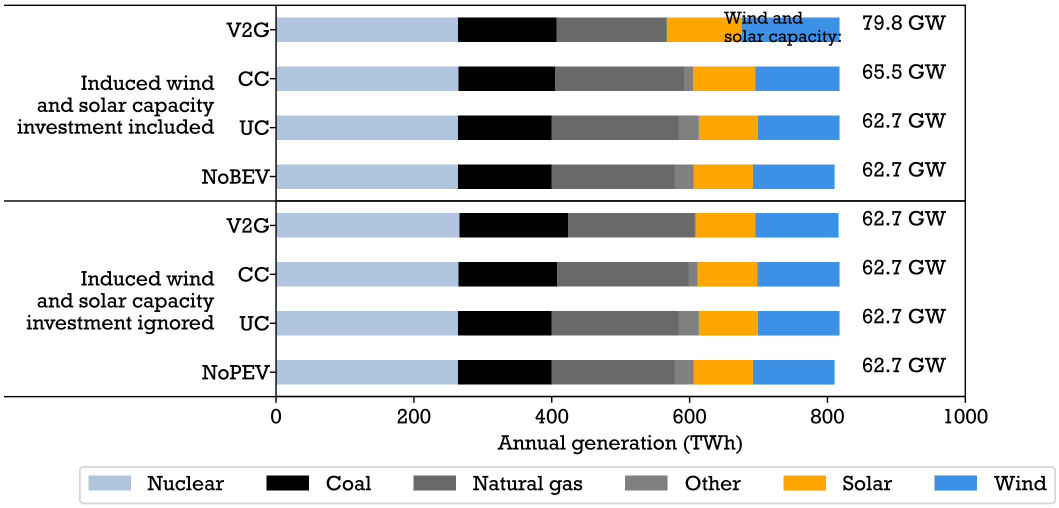

Figure S19: Annual generation of different fuel types, by different PEV and PEV charging intervention scenarios and analysis approaches. Wind and solar capacity varies across PEV and PEV charging intervention scenarios in wind and solar capacity investment scenarios, as PEV and PEV charging interventions affect the maximum profitable wind and solar capacity installations. 'Natural gas' fuel type includes combined cycle natural gas generators and gas turbine generators. 'Other' fuel types include biomass, fossil waste, fuel cell, hydro, landfill gas, municipal solid waste, non-fossil waste, and oil or gas steam.

Table S7: Annual emissions and environmental externalities of greenhouse gases and air pollutants, by different grid intervention scenarios and analysis approaches. wind and power capacity varies across grid intervention scenarios in wind and power scenarios, as PEV charging intervention intervention affects the maximum profitable wind and power capacity installation. Environmental externalities for GHG emissions are calculated with social cost of carbon of \$204/ton CO_2e. The AP3 model is used to calculate those of pollutants. All costs are in 2024 USD.

			Emission Externalities ($MM)					
			GHG	NOx	SO2	NH3	PM25	VOC
Wind and Solar Capacity Investment	Ignored	NoPEV	50455	1765	4195	165	1208	14
		Uncontrolled charging	51152	1771	4206	182	1246	15
		Cost-minimizing charging	50895	1685	4380	181	1199	13
		V2G	51467	1730	4595	180	1182	13
	Included	NoPEV	50455	1765	4195	165	1208	14
		Uncontrolled charging	51152	1771	4206	182	1246	15
		Cost-minimizing charging	50013	1662	4286	178	1180	13
		V2G	46155	1568	4128	163	1052	12

References

[1] S. P. Holland, M. J. Kotchen, E. T. Mansur, and A. J. Yates, "Why marginal CO2 emissions are not decreasing for US electricity: Estimates and implications for climate policy," *Proceedings of the National Academy of Sciences*, vol. 119, no. 8, e2116632119, Feb. 22, 2022, Publisher: Proceedings of the National Academy of Sciences. DOI: 10.1073/pnas.2116632119. [Online]. Available: https://www.pnas.org/doi/abs/10.1073/pnas.2116632119 (visited on 02/03/2024).

[2] R. Tu, Y. (Gai, B. Farooq, D. Posen, and M. Hatzopoulou, "Electric vehicle charging optimization to minimize marginal greenhouse gas emissions from power generation," *Applied Energy*, vol. 277, p. 115 517, Nov. 1, 2020, ISSN: 0306-2619. DOI: 10.1016/j.apenergy.2020.115517. [Online]. Available: http://www.sciencedirect.com/science/article/pii/S0306261920310291 (visited on 10/04/2020).

[3] J. Chen, F. Wang, X. He, X. Liang, J. Huang, S. Zhang, and Y. Wu, "Emission mitigation potential from coordinated charging schemes for future private electric vehicles," *Applied Energy*, vol. 308, p. 118 385, Feb. 15, 2022, ISSN: 0306-2619. DOI: 10.1016/j.apenergy.2021.118385. [Online]. Available: https://www.sciencedirect.com/science/article/pii/S0306261921016238 (visited on 03/22/2022).

[4] M. Wang and M. T. Craig, "The value of vehicle-to-grid in a decarbonizing california grid," *Journal of Power Sources*, vol. 513, p. 230 472, Nov. 30, 2021, ISSN: 0378-7753. DOI: 10.1016/j.jpowsour.2021.230472. [Online]. Available: https://www.sciencedirect.com/science/article/pii/S0378775321009757 (visited on 07/01/2023).

[5] R. Shi, S. Li, P. Zhang, and K. Y. Lee, "Integration of renewable energy sources and electric vehicles in v2g network with adjustable robust optimization," *Renewable Energy*, vol. 153, pp. 1067–1080, Jun. 1, 2020, ISSN: 0960-1481. DOI: 10.1016/j.renene.2020.02.027. [Online]. Available: https://www.sciencedirect.com/science/article/pii/S0960148120302135 (visited on 04/25/2024).

[6] N. A. Ryan, J. X. Johnson, and G. A. Keoleian, "Comparative assessment of models and methods to calculate grid electricity emissions," *Environmental Science & Technology*, vol. 50, no. 17, pp. 8937–8953, Sep. 6, 2016, Publisher: American Chemical Society, ISSN: 0013-936X. DOI: 10.1021/acs.est.5b05216. [Online]. Available: https://doi.org/10.1021/acs.est.5b05216 (visited on 03/05/2024).

[7] X. Chen, H. Zhang, Z. Xu, C. P. Nielsen, M. B. McElroy, and J. Lv, "Impacts of fleet types and charging modes for electric vehicles on emissions under different penetrations of wind power," *Nature Energy*, vol. 3, no. 5, pp. 413–421, 2018, Publisher: Nature Publishing Group.

[8] J. Owens, I. Miller, and E. Gençer, "Can vehicle-to-grid facilitate the transition to low carbon energy systems?" *Energy Advances*, vol. 1, no. 12, pp. 984–998, 2022, Publisher: Royal Society of Chemistry. DOI: 10.1039/D2YA00204C. [Online]. Available: https://pubs.rsc.org/en/content/articlelanding/2022/ya/d2ya00204c (visited on 06/20/2023).

[9] P. Gagnon and W. Cole, "Planning for the evolution of the electric grid with a long-run marginal emission rate," *iScience*, vol. 25, no. 3, p. 103 915, Mar. 18, 2022, ISSN: 2589-0042. DOI: 10.1016/j.isci.2022.103915. [Online]. Available: https://www.sciencedirect.com/science/article/pii/S2589004222001857 (visited on 02/12/2024).

[10] T. Brown, D. Schlachtberger, A. Kies, S. Schramm, and M. Greiner, "Synergies of sector coupling and transmission reinforcement in a cost-optimised, highly renewable european energy system," *Energy*, vol. 160, pp. 720–739, Oct. 1, 2018, ISSN: 0360-5442. DOI: 10.1016/j.energy.2018.06.222. [Online]. Available: https://www.sciencedirect.com/science/article/pii/S036054421831288X (visited on 02/12/2024).

[11] F. Manríquez, E. Sauma, J. Aguado, S. de la Torre, and J. Contreras, "The impact of electric vehicle charging schemes in power system expansion planning," *Applied Energy*, vol. 262, p. 114 527, Mar. 15, 2020, ISSN: 0306-2619. DOI: 10.1016/j.apenergy.2020.114527. [Online]. Available: https://www.sciencedirect.com/science/article/pii/S0306261920300398 (visited on 02/12/2024).

[12] A. Weis, P. Jaramillo, and J. Michalek, "Estimating the potential of controlled plug-in hybrid electric vehicle charging to reduce operational and capacity expansion costs for electric power systems with high wind penetration," *Applied Energy*, vol. 115, pp. 190–204, Feb. 15, 2014, ISSN: 0306-2619. DOI: 10.1016/j.apenergy.2013.10.017. [Online]. Available: https://www.sciencedirect.com/science/article/pii/S0306261913008374 (visited on 11/27/2021).

[13] E. S. Hittinger and I. M. L. Azevedo, "Bulk energy storage increases united states electricity system emissions," *Environmental Science & Technology*, vol. 49, no. 5, pp. 3203–3210, Mar. 3, 2015, Publisher: American Chemical Society, ISSN: 0013-936X. DOI: 10.1021/es505027p. [Online]. Available: https://doi.org/10.1021/es505027p (visited on 04/27/2024).

[14] M. Muratori, "Impact of uncoordinated plug-in electric vehicle charging on residential power demand," *Nature Energy*, vol. 3, no. 3, pp. 193–201, 2018, Publisher: Nature Publishing Group.

[15] M. E. Kahn and R. K. Vaughn, "Green market geography: The spatial clustering of hybrid vehicles and LEED registered buildings," *The B.E. Journal of Economic Analysis & Policy*, vol. 9, no. 2, Mar. 30, 2009, Publisher: De Gruyter, ISSN: 1935-1682. DOI: 10.2202/1935-1682.2030. [Online]. Available: https://www.degruyter.com/document/doi/10.2202/1935-1682.2030/html (visited on 07/01/2023).

[16] P. R. Brown and F. M. O'Sullivan, "Spatial and temporal variation in the value of solar power across united states electricity markets," *Renewable and Sustainable Energy Reviews*, vol. 121, p. 109 594, Apr. 1, 2020, ISSN: 1364-0321. DOI: 10.1016/j.rser.2019.109594. [Online]. Available: https://www.sciencedirect.com/science/article/pii/S1364032119308020 (visited on 07/01/2023).

[17] M. Vanatta, M. T. Craig, B. Rathod, J. Florez, I. Bromley-Dulfano, and D. Smith, "The costs of replacing coal plant jobs with local instead of distant wind and solar jobs across the united states," *iScience*, vol. 25, no. 8, p. 104 817, Aug. 2022, ISSN: 25890042. DOI: 10.1016/j.isci.2022.104817. [Online]. Available: https://linkinghub.elsevier.com/retrieve/pii/S2589004222010896 (visited on 07/01/2023).

[18] M. Carrión and R. Zárate-Miñano, "Operation of renewable-dominated power systems with a significant penetration of plug-in electric vehicles," *Energy*, vol. 90, pp. 827–835, Oct. 1, 2015, ISSN: 0360-5442. DOI: 10.1016/j.energy.2015.07.111. [Online]. Available: https://www.sciencedirect.com/science/article/pii/S0360544215010063 (visited on 06/25/2023).

[19] A. Jenn, K. Clark-Sutton, M. Gallaher, and J. Petrusa, “Environmental impacts of extreme fast charging,” *Environmental Research Letters*, vol. 15, no. 9, p. 094 060, Aug. 2020, Publisher: IOP Publishing, ISSN: 1748-9326. DOI: 10.1088/1748-9326/ab9870. [Online]. Available: https://dx.doi.org/10.1088/1748-9326/ab9870 (visited on 04/29/2024).

[20] A. Weis, J. J. Michalek, P. Jaramillo, and R. Lueken, “Emissions and cost implications of controlled electric vehicle charging in the u.s. PJM interconnection,” *Environmental Science & Technology*, vol. 49, no. 9, pp. 5813–5819, May 5, 2015, ISSN: 0013-936X, 1520-5851. DOI: 10.1021/es505822f. [Online]. Available: https://pubs.acs.org/doi/10.1021/es505822f (visited on 10/06/2020).

[21] P. Nunes and M. C. Brito, “Displacing natural gas with electric vehicles for grid stabilization,” *Energy*, vol. 141, pp. 87–96, Dec. 15, 2017, ISSN: 0360-5442. DOI: 10.1016/j.energy.2017.09.064. [Online]. Available: https://www.sciencedirect.com/science/article/pii/S0360544217315852 (visited on 07/01/2023).

[22] K. E. Forrest, B. Tarroja, L. Zhang, B. Shaffer, and S. Samuelsen, “Charging a renewable future: The impact of electric vehicle charging intelligence on energy storage requirements to meet renewable portfolio standards,” *Journal of Power Sources*, vol. 336, pp. 63–74, Dec. 30, 2016, ISSN: 0378-7753. DOI: 10.1016/j.jpowsour.2016.10.048. [Online]. Available: https://www.sciencedirect.com/science/article/pii/S0378775316314409 (visited on 06/25/2023).

[23] B. Tarroja, L. Zhang, V. Wifvat, B. Shaffer, and S. Samuelsen, “Assessing the stationary energy storage equivalency of vehicle-to-grid charging battery electric vehicles,” *Energy*, vol. 106, pp. 673–690, Jul. 1, 2016, ISSN: 0360-5442. DOI: 10.1016/j.energy.2016.03.094. [Online]. Available: https://www.sciencedirect.com/science/article/pii/S0360544216303383 (visited on 08/13/2025).

[24] PJM, “Energy transition in PJM: Frameworks for analysis,” Dec. 15, 2021. [Online]. Available: https://pjm.com/-/media/committees-groups/committees/mrc/2021/20211215/20211215-item-09-energy-transition-in-pjm-whitepaper.ashx.

[25] L. Vimmerstedt, T. Stehly, S. Akar, A. Sekar, B. Mirletz, D. Stright, C. Augustine, P. Beiter, P. Bhaskar, N. Blair, S. Cohen, W. Cole, P. Duffy, D. Feldman, P. Gagnon, P. Kurup, C. Murphy, V. Ramasamy, J. Robins, J. Zuboy, D. Oladosu, and J. Hoffmann, “2022 annual technology baseline (ATB) cost and performance data for electricity generation technologies,” DOE Open Energy Data Initiative (OEDI); National Renewable Energy Laboratory (NREL), 5716, Jun. 1, 2022. DOI: 10.25984/1871952. [Online]. Available: https://www.osti.gov/dataexplorer/biblio/dataset/1871952 (visited on 07/23/2023).

[26] R. Lueken and J. Apt, “The effects of bulk electricity storage on the PJM market,” *Energy Systems*, vol. 5, no. 4, pp. 677–704, Dec. 1, 2014, ISSN: 1868-3975. DOI: 10.1007/s12667-014-0123-7. [Online]. Available: https://doi.org/10.1007/s12667-014-0123-7 (visited on 07/20/2023).

[27] M. Bruchon, Z. L. Chen, and J. Michalek, “Cleaning up while changing gears: The role of battery design, fossil fuel power plants, and vehicle policy for reducing emissions in the transition to electric vehicles,” *Environmental Science & Technology*, vol. 58, no. 8, pp. 3787–3799, Feb. 27, 2024, Publisher: American Chemical Society, ISSN: 0013-936X. DOI: 10.1021/

acs.est.3c07098. [Online]. Available: https://doi.org/10.1021/acs.est.3c07098 (visited on 05/08/2024).

[28] A. Weis, P. Jaramillo, and J. Michalek, “Consequential life cycle air emissions externalities for plug-in electric vehicles in the PJM interconnection,” *Environmental Research Letters*, vol. 11, no. 2, p. 024 009, Feb. 2016, Publisher: IOP Publishing, ISSN: 1748-9326. DOI: 10.1088/1748-9326/11/2/024009. [Online]. Available: https://dx.doi.org/10.1088/1748-9326/11/2/024009 (visited on 04/25/2024).

[29] B. Sergi and W. Cole, “Operating reserves in ReEDS,” National Renewable Energy Lab. (NREL), Golden, CO (United States), NREL/PR-6A40-81706, Dec. 1, 2021. DOI: 10.2172/1841961. [Online]. Available: https://www.osti.gov/biblio/1841961 (visited on 07/26/2023).

[30] M. T. Craig, P. Jaramillo, H. Zhai, and K. Klima, “The economic merits of flexible carbon capture and sequestration as a compliance strategy with the clean power plan,” *Environmental Science & Technology*, vol. 51, no. 3, pp. 1102–1109, Feb. 7, 2017, Publisher: American Chemical Society, ISSN: 0013-936X. DOI: 10.1021/acs.est.6b03652. [Online]. Available: https://doi.org/10.1021/acs.est.6b03652 (visited on 07/26/2023).

[31] “EPA report on the social cost of greenhouse gases: Estimates incorporating recent scientific advances,” Nov. 2023.
